# Iron and Copper Intracellular Chelation as an Anticancer Drug Strategy

**DOI:** 10.3390/inorganics6040126

**Published:** 2018-11-30

**Authors:** Kavita Gaur, Alexandra M. Vázquez-Salgado, Geraldo Duran-Camacho, Irivette Dominguez-Martinez, Josué A. Benjamín-Rivera, Lauren Fernández-Vega, Lesly Carmona Sarabia, Angelys Cruz García, Felipe Pérez-Deliz, José A. Méndez Román, Melissa Vega-Cartagena, Sergio A. Loza-Rosas, Xaymara Rodriguez Acevedo, Arthur D. Tinoco

**Affiliations:** 1 Department of Chemistry, University of Puerto Rico, Río Piedras Campus, Río Piedras, PR 00931, USA; 2 Department of Biology, University of Puerto Rico, Río Piedras Campus, Río Piedras, PR 00931, USA

**Keywords:** copper and iron chelators in cancer, transmetalation, metastasis, angiogenesis, metallomics

## Abstract

A very promising direction in the development of anticancer drugs is inhibiting the molecular pathways that keep cancer cells alive and able to metastasize. Copper and iron are two essential metals that play significant roles in the rapid proliferation of cancer cells and several chelators have been studied to suppress the bioavailability of these metals in the cells. This review discusses the major contributions that Cu and Fe play in the progression and spreading of cancer and evaluates select Cu and Fe chelators that demonstrate great promise as anticancer drugs. Efforts to improve the cellular delivery, efficacy, and tumor responsiveness of these chelators are also presented including a transmetallation strategy for dual targeting of Cu and Fe. To elucidate the effectiveness and specificity of Cu and Fe chelators for treating cancer, analytical tools are described for measuring Cu and Fe levels and for tracking the metals in cells, tissue, and the body.

## 1. Introduction

In the U.S. cancer is second only to heart disease for the leading causes of death and is soon expected to become the number one cause [1]. The gold standard in the treatment of cancer remains the traditional approaches, surgery, radiation, and older drugs like cisplatin (and its second generation of compounds) but their success rates are extremely low and their applicability is limited [2]. Newer drugs, both broad and narrow spectrum, suffer from off target side effects and toxicity due to their lack of specificity for cancer cells and acquired cellular resistance. The omics-era of cancer research has brought a wealth of information regarding molecular details of cancer cells, the different types of cancer cells, the differences in the cause of the disease, and has also revealed new drug targets [3,4]. There is now much potential for personalized treatment especially in the field of immunotherapeutics [5-7], which seek to bolster the body’s natural immune defense mechanism against aberrant cells.

A very promising direction in the development of anticancer drugs is inhibiting the molecular pathways that keep cancer cells alive and able to metastasize. Much attention has been directed toward copper (Cu) and iron (Fe) because they are at the root of cancer cell rapid proliferation and metastasis. Several Cu(I)/Cu(II)and Fe(II)/Fe(III) chelators, originally designed for other applications, are being repurposed as anticancer agents [8,9]. The chelators of interest operate by different proposed mechanisms including binding the metals extracellularly, depleting the metal ions from cancer cells or from intracellular sites that regulate cell function, or inhibiting molecular pathways without necessarily removing them. The common thread in all of these mechanisms is decreasing the bioavailability of functional forms of these metals. Using Cu and Fe chelators for anticancer treatment is extremely promising and several chelators are currently in anticancer clinical trials [10]. In this review article we discuss the roles of Cu and Fe in the progression and spreading of cancer. We also examine pertinent Cu and Fe chelators as potential anticancer drugs and describe efforts to improve their cellular uptake, efficacy, and exclusive cancer cell responsiveness. Transmetallation is introduced as a strategy to overcome the limitations inherent in the chelators binding other metals by manipulating coordination chemistry to target Cu and Fe chelation while releasing a cytotoxic metal into cancer cells. Finally, we briefly examine analytical tools that can be used to gauge the effectiveness of Cu and Fe chelators in inhibiting cancer growth and in targeting the cancer itself.

## 2. The Role of Cu in Cancer

### 2.1. Cu as an Important Biological Co-Factor

Cu is an important metal in many biological processes and is essential for cellular physiology. The concentration of Cu in blood is around 5 to 25 μM bound majorly with ceruloplasmin and other Cu binding proteins [11]. Its function stems from Cu’s potent redox activity because it can easily change between Cu(II) and Cu(I) in the biological environment [11]. Grubman et al. describe that Cu, like calcium (Ca) and zinc (Zn), can play a modulatory role in cell signal transduction pathways [11]. Cu is an important biological co-factor of many essential enzymes, cuproenzymes, that take part in a variety of cellular processes in mammals [12]. With Cu as a co-factor, these cuproenzymes can be involved in different enzymatic functions, such as melanin synthesis (tyrosinase), energy metabolism (cytochrome *c* oxidase), tissue synthesis (lysil oxidase), antioxidative activity [Cu zinc superoxide dismutase (SOD1)], dopamine synthesis (dopaminβ-hydroxylase) and Fenton reactions, in which the potent redox activity of which can produce reactive oxygen species (ROS) [11]. Cu transportation into cells is regulated by the Cu transporter 1 (Ctr1) membrane protein when Cu(II) is reduced to Cu(I) by reductases. The metal is then transferred to Cu chaperones (one of which is ATOX1), and subsequently delivered to several organelles inside the cell. Cu can bind to Cu-dependent proteins in the Golgi apparatus, such as antioxidants to prevent ROS formation by free Cu ions. Some of the Cu forms a labile Cu(I) pool [13]. As a way of regulating these pathways and high levels of Cu, the Cu importer is internalized and degraded [14].

**Figure 1.**
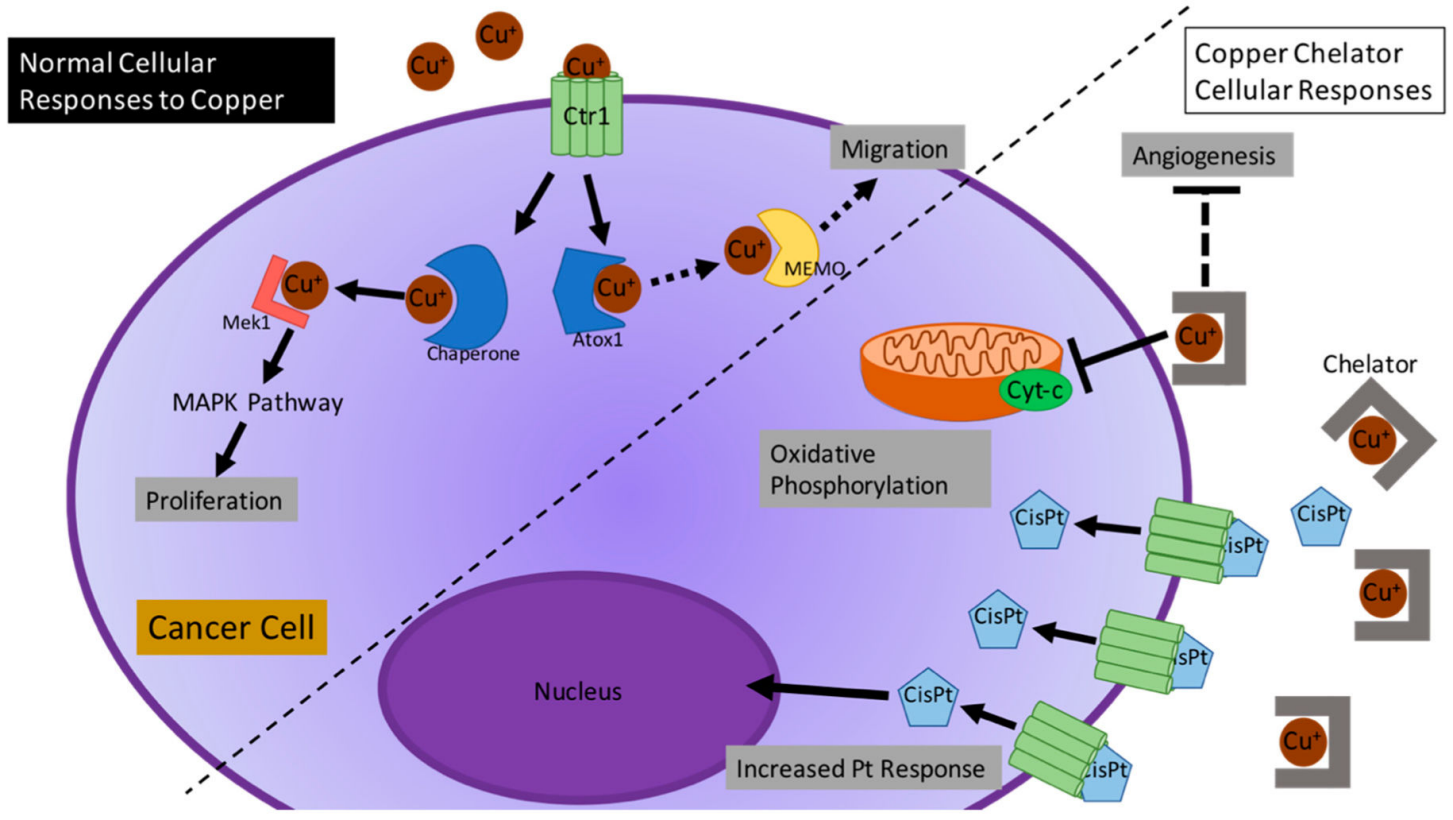
Normal cellular responses to Cu and altered cellular responses with Cu chelator treatment in cancer cells. Once Cu(II) is reduced to Cu(I), it can enter the cell through the high-affinity Cu transporter Ctr1. Cu(I) can be bound to Cu chaperones and transported in the cytoplasm to several organelles, not shown here [14,23,24]. Cu can be delivered to many Cu-binding proteins, including Mek1 and MEMO. Mek1, when activated by Cu binding, will activate the MAPK pathway, which leads to cell proliferation. Antioxidant 1 Cu chaperone (ATOX1) mediates the delivery of Cu to Cu-binding proteins located in the lamellipodia (not shown here), such as MEMO, which is separately known to regulate cancer cell migration and could have implications in metastasis [12,24]. Cu has been associated with angiogenesis [23]. Cu chelator tetrathiomolybdate (TM) treatment can delay the activation of angiogenesis on pancreatic islets. In pancreatic neuroendocrine tumor cells, treatment with TM reduced the activity of Cyt-c, which results in decreased oxidative phosphorylation [23]. CisPt can enter cancer cells through Ctr1. When Cu is present in high levels (a common property of many cancers) cells downregulate the expression of Ctr1 [12]. This results in less CisPt entering the cells. Here, we show the use of Cu chelators to overcome CisPt resistance and make cancer cells more sensitive to this chemotherapeutic agent [12,14,15,23-25].

**Figure 2.**
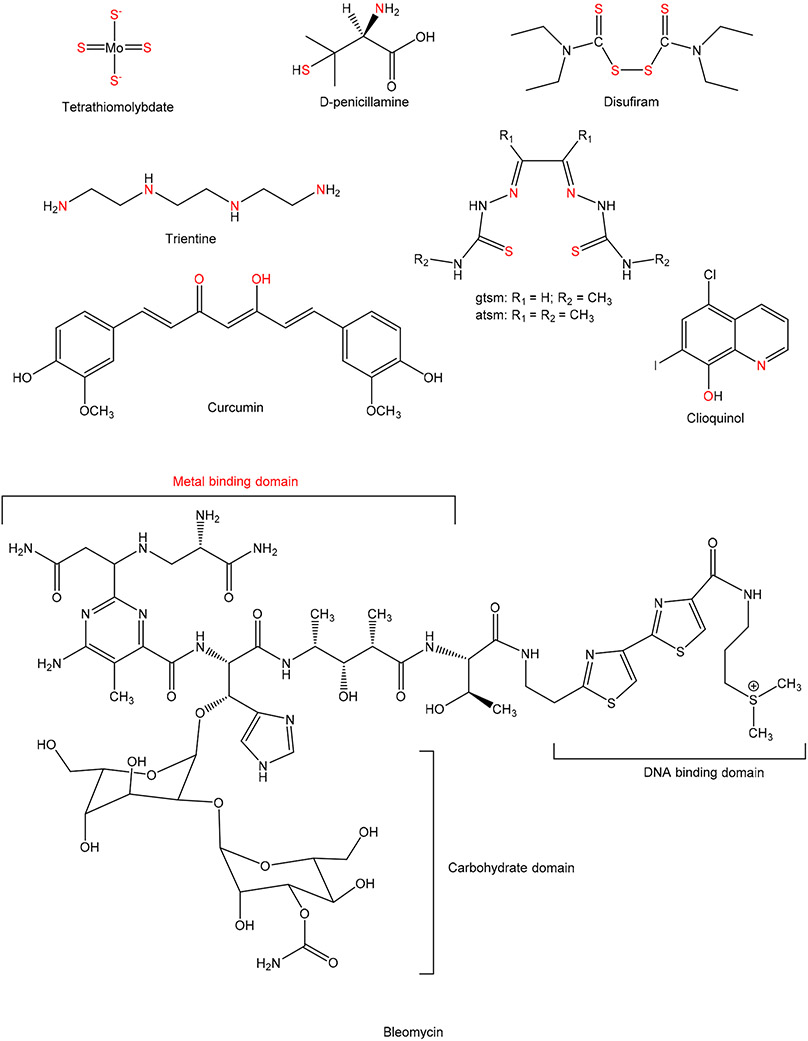
The structure of select Cu chelators for potential anticancer applications.

Changes in the homeostasis of Cu play an important role in different types of diseases, such as inflammation, neurodegeneration, and cancer. In cancer, Cu is involved in several aspects, including metastasis and angiogenesis [11]. Figure 1 shows the role of Cu in a normal cell and in a cancer cell. During angiogenesis, a hallmark of cancer progression, it has been demonstrated that reduced levels of Cu also decreases COX1 and ATP levels. At the same time, oxidative phosphorylation is reduced, which results in decreased growth of proliferative cancer cells [15]. Depletion of Cu was found to inhibit the epithelial-to-mesenchymal transition (EMT). EMT describes the process in which cells lose polarity and cell-cell adhesion. Hence, chelation of Cu can potentially reduce the migratory and invasive properties of cancer cells [11,16]. Different Cu chelators are used in the treatment of Wilson disease, including D-penicillamine (D-Pen), tetrathiomolybdate (TM), and trientine (Trien) (Figure 2), which can modulate cell invasiveness [17,18]. Recent studies demonstrate that small molecules can specifically target the Cu chaperone for superoxide dismutase (CCS) and antioxidant 1 Cu chaperone (ATOX1) and inhibit the cellular Cu transport [19]. ATOX1 on the other hand, plays a major role in Cu homeostasis and it has been seen that its deficiency may lead to the accumulation of high levels of intracellular Cu, and the cause was found impaired with the efflux of cellular Cu [20]. Barresi el al also demonstrated that ATOX1 deficiency could induce copper dyshomeostasis [21]. In the study, they used small Cu chelator and ionophore (binds and transports the metal ions into the cells) molecules to analyze the impact of silencing ATOX1 in colon carcinoma (Caco-2) cells. They observed that the Cu ionophore 5-chloro-8-hydroxyquinoline (ClHQ), was able to exhibit Cu-dependent toxicity in Caco-2 cells and the toxicity was enhanced by ATOX1 silencing. Conversely the Cu chelator *N,N,N′,N′*-tetrakis(2-pyridylmethyl)ethylenediamine (TPEN) generates toxicity which was reversed by the addition of Cu(II), consistent with its ability to remove metal ions. They also observed increased Caco-2 cells sensitivity to TPEN upon gene silencing of ATOX1 [21].

### 2.2. Current Studies of Cu in Different Cancer-Altered Biological Processes

In the previous section, we introduced Cu as a metal involved in angiogenesis and cancer metastasis. Here, we dig deeper on the role of Cu in these cancer-altered biological processes, including cell growth and survival. During the last several decades, it has been discovered that Cu plays a role in the development and progression of cancer. The mechanisms by which it does are being explored. Cu has not yet been found to help in the normal-to-cancer cell transformation, but it plays a role in the development of new blood vessels called angiogenesis and the spreading of malignant tumors to secondary sites called metastasis. Ding et al. evaluated the functions of CD147, a signaling receptor in the signaling pathway of extracellular divalent Cu in hepatocellular carcinoma cells (HCC). Their data show that Cu(II)can bind to the extracellular membrane-proximal domain of CD147. This binding activates the PI3K/Akt signaling pathway, important for cell survival and growth, causing CD147 to self-dissociate. At the same time, they noticed the up-regulation of matrix metalloproteinases MMP-2 and MMP-14 in HCC cells. MMP-2 up-regulation induces cell invasion by stimulating neighboring fibroblasts in the presence of Cu(II). Without CD147, extracellular Cu(II) cannot affect the regulation of metalloproteinases. The action of CD147, however, cannot induce the production of MMPs. Ding et al. associated the action of extracellular Cu(II) to the function of CD147 as an interdependent process [22].

Animal studies have demonstrated that Cu-chelating drugs show antiangiogenic effects. The mechanisms by which Cu regulates angiogenesis are unknown. Ishida et al. assessed the effects of Cu chelation with tetrathiomolybdate (Figure 2) on the formation of angiogenic islets [23]. They noticed that the number of angiogenic islets decreased significantly after treatment with TM during premalignant lesions, but not during the formation of tumors and their subsequent growth suggesting that TM treatment can delay the angiogenic switch or the “activation” of angiogenesis [23]. Ishida et al. also demonstrated that elevated levels of Cu in drinking water increased the proliferation rate of cancer cells. They determined that intracellular Cu regulated the activity of cytochrome *c* oxidase (CcO) in cancer cells. CcO forms the last complex of the electron transport chain to produce ATP during oxidative phosphorylation. Depletion of Cu by TM decreases CcO activity in pancreatic neuroendocrine tumor cells (βTC3) thereby reducing oxidative phosphorylation in cancer cells. For ATP production under Cu-limited conditions, they proved that cells rely on glycolysis. These findings suggest new target strategies for cancer treatment where Cu chelators can be tested in combination with classical chemotherapy (platinum-based drugs) or with a glycolysis inhibitor, which would potentially eliminate overall production of ATP in cancer cells [23].

For cancer metastasis to occur, many signaling pathways and processes dependent on cell motility and migration must first occur. Cells move through the formation of protrusion “toes” at the leading edge, known as lamellipodia and filopodia [12]. Several Cu-dependent proteins have been implicated in cancer metastasis. ATOX1 is a Cu chaperone that picks up Cu from the Ctr1 and delivers it to ATPases found in the Golgi Apparatus [12]. It has not been determined if ATOX1 plays a direct role in Cu-dependent cancer metastasis. Blockhuys et al. show the presence of ATOX1 in the nucleus (acting as a transcription factor) and cytoplasm, but more interestingly, in the lamellipodia of MCF-7 and MDA-MB-231 breast cancer cell lines. ATOX1 silencing with siRNAs did not affect the formation of lamellipodium in MDA-MB-231 cells. Nevertheless, using a wound-healing assay, they determined that ATOX1 is required for breast cancer cell migration in vitro. ATOX1 may be mediating the delivery of Cu to Cu-binding proteins located in the lamellipodia, such as MEMO (Mediator of ErbB2-driven cell motility), which is separately known to regulate cancer cell migration. Another study demonstrated similar results [26], that ATOX1 was being delivered to the lamellipodia of vascular smooth muscle cells and that it was essential for platelet-derived growth factor-mediated migration.

McDonald et al. evaluated the behavior of MEMO as a Cu-dependent redox protein in the process of metastasis and migration [27]. Studying breast cancer, they found that MEMO acts in the migration and invasion of cancer cells in vitro promoting a more oxidized environment and that MEMO stimulates the production of ROS. In vivo studies in 6-week-old female non-obese diabetic/severe combined immunodeficient mice demonstrated that the activity of MEMO was present in the spontaneous metastasis of breast cancer to the lungs. The authors found that the abundance of MEMO can be correlated as an independent factor of early distant metastasis [27].

Recent studies examine the role of Cu in many biological processes that are commonly altered in cancer development and progression. Cu is a co-factor of enzymes that participates in several biochemical processes, such as oxidative phosphorylation. Turski et al. found that the MAPK (mitogen activated protein kinase) signaling pathway can be stimulated by the high-affinity Cu transporter Ctr1, which is overexpressed in genetically engineered mice models (GEMMs) of human cervical carcinoma when the Ras pathway is activated by external growth factors [23,24]. The Ras protein is a GTPase encoded by the Ras oncogene, which can subsequently activate several oncogenic signaling pathways, including the MAPK pathway. This pathway is involved in regulating cell proliferation, survival, motility, and metabolism, all of which can be altered in cancer cells. Even more, mutations that hyperactivate molecular components of the MAPK pathway show direct association with the development and progression of cancer. Tuski et al. found that reduction of intracellular Cu, using a Cu chelator, results in decreased activation of the MAPK pathway [24]. At a molecular level, they found that Cu binds to Mek1, a component of the said pathway, which phosphorylates Erk [28]. Their research with in vitro cell culture and fly and mice models demonstrated that Cu-dependent Ctr1 affects MAPK pathway activation.

In a related finding, increased levels of Ctr1 mRNA in colorectal cancer resulted in a parallel increase in transcript levels for Cu efflux pump ATP7A, copper metabolism Murr1 domain containing 1 (COMMD1), the cytochrome *c* oxidase assembly factors (synthesis of cytochrome *c* oxidase 1 (SCO1) and cytochrome c oxidase Cu chaperone 11 (COX11)), the cupric reductase enzyme six transmembrane epithelial antigen of the prostate (STEAP3), and the metal-regulatory transcription factors (MTF1, MTF2) and specificity protein 1 (SP1) [29]. This data suggests that due to the high proliferation rates, cancer cells require a greater amount of Cu than the normal cells. Considering that many cancer patients’ serum and cancer cells possess increased levels of Cu, these results have strong implications on the potential role of Cu on proliferation and metastasis [30].

BRAF (V6ooE) is a kinase that phosphorylates Mek1, which results in the downstream signaling activation of the MAPK pathway. In a study, half of the melanoma patients showed mutations on the BRAF^V6ooE^ gene. Current drug treatments for BRAF^V6ooE^-positive melanoma focus on administering inhibitors for BRAF and Mek1/2 but as with many other drugs, subsets of melanomas develop resistance. Due to the Cu-dependence of Mek1, downregulating Ctr1 (Cu transporter) has been proposed as a treatment option. Nevertheless, melanoma cells counteract this by producing Cu-independent Mek1. Brady et al. demonstrated that the use of TM, which was tested in vivo by Ishida et al., reduces the levels of Cu and activation of the MAPK pathway, resulting in the inhibition of BRAF^V6ooE^-positive melanoma tumor cell growth of cells resistant to BRAF and Mek inhibitors. Other findings of this study showed toxicities in GEMMs for melanoma, but increased survival benefits after treatment with TM alone or in combination with a BRAF inhibitor, vemurafenib. This study highlights the potential of TM as a treatment for BRAF^V6ooE^-positive melanoma patients alone or in combination with other melanoma treatments [25].

Altogether, these findings highlight Cu’s role in cancer progression, metastasis, and angiogenesis and support the study of Cu chelators as potential cancer therapy alone or combined with other treatments already approved or in cancer clinical trials. We have shown the importance of studying Cu-dependent proteins, such as MEMO and signaling receptor CD147 and their involvement in migration and metastasis processes.

### 2.3. Chelation Strategies

**Figure 3.**
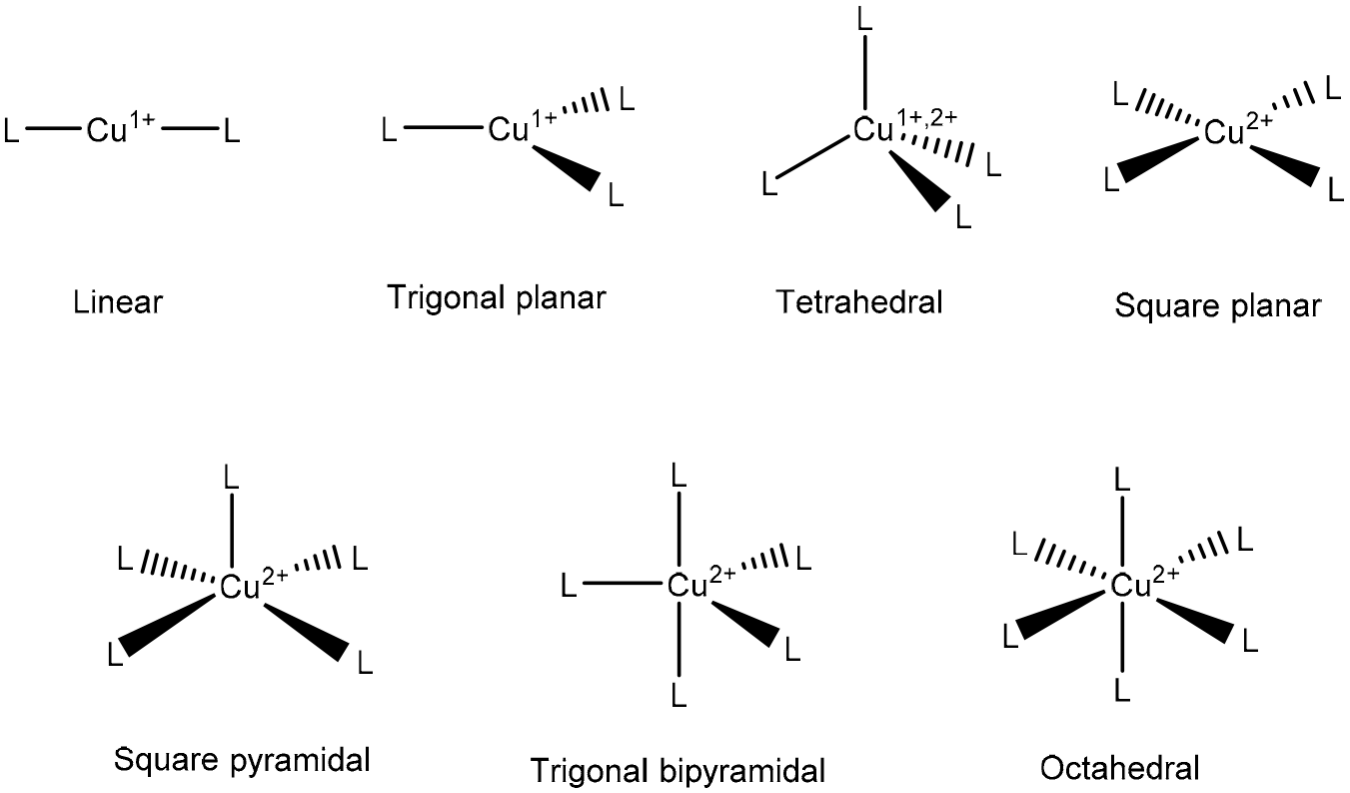
Typical geometries for Cu(I) and Cu(II) chelation.

Several strategies to target Cu for cancer therapy are the use of Cu chelators and Cu ionophores. By definition, chelators are the compounds that sequester the metal ions and can make them bio-unavailable, in contrast, ionophores usually elevate intracellular bioavailability of metal ions [31,32]. Features of both chelators (intracellular chelation) and ionophores (extracellular chelation) can be utilized in anticancer drug designing strategy [33]. Denoyer et al. assessed recent literature on the effects of Cu transporters in cells resistant against platinum complexes. Cisplatin (CisPt) and other platinum-based drugs can enter cells through Ctr1. When Cu is present in high levels (a common property of many cancers) cells downregulate the expression of Ctr1. This results in less CisPt entering the cells [14]. The use of Cu chelators to overcome this resistance has become more popular (Figure 1). Cu chelators were first synthesized for Wilson’s disease patients that have Cu overload usually localized to the liver. Clinical data is variable between cancer types, but there seems to be a common behavior of Cu chelation being most useful in preventing cancer progression of micrometastasis, where angiogenesis has not reached later stages yet, as demonstrated by Ishida et al. [23]. This suggests that the role of Cu in cancer progression is mainly due to facilitating angiogenesis. Cu chelators could play important functions in inhibiting Cu dependent pathways for cancer progression. Cu(I) is a soft Lewis Acid that is typically bound by the S donor atoms and intermediate N donor atoms in ligands. It tends to form coordination compounds with coordination numbers 2, 3, and 4 (Figure 3) [34]. Cu(II) is an intermediate Lewis Acid that in coordination compounds is found bound to N donor atoms and hard O atoms. Cu(II) coordination compounds are very commonly of coordination numbers 4, 5, and 6 (Figure 3) [34]. Due to the reducing environment of cells, Cu dominantly exists intracellularly in the +1 oxidation state as opposed to the +2 form in blood. In the following, we discuss a selection of Cu chelators for anticancer application (Figure 2). Several of these ligands have the ability to bind Cu in both the +1 and +2 oxidation states and so are capable of binding Cu(II) extracellularly and Cu(I) intracellularly.

#### 2.3.1. D-Penicillamine

D-Pen is a D-isomer of dimethylcysteine which is used for the treatment of Wilson’s disease [35]. Additionally, it is used for the treatment of rheumatoid arthritis, Cu poisoning, and lead poisoning [36,37]. D-pen was developed by Walshe in 1956 and sold under the trade name of Cuprimine and Depen. It is an α-amino acid metabolite of penicillin which was discovered by Walshe from the urine of patients with liver disease and treated with penicillin [38]. Later, he isolated the compound and identified its Cu chelating properties. D-Pen (ββ dimethyl cysteine) is a thiol that contains a sulfhydryl group which binds to Cu(I) and eliminates it via urine both in normal people and in patients with Wilson’s disease [35]. Depending on the number of functional groups involved in the formation of the complex, D-pen can function as a monodentate, bidentate, or even tridentate ligand [39]. Walshe et al. proposed the possibilities of the bond formation between Cu(I) and D-pen, (i) a single Cu could bind to a single thiol group (C─S─Cu^+^), (ii) a Cu atom might be involved in binding with the thiol groups of two D-pen molecules on both sides (─S─Cu─S─), or (iii) a ring compound might be formed by the linkage of one Cu atom with the thiol and amino groups of one D-pen [35]. The pKa of the thiol group of D-pen is 7.9 which makes it more reactive thus the degree of ionization for D-pen at physiological pH would be higher than that compared to cysteine (pKa 8.3) and Glutathione (GSH; pKa 8.8) [40]. Moreover, it has been seen that D-pen oxidizes at higher rates in the presence of Cu compared to other thiols (e.g., GSH, cysteine, Homocysteine, *N*-acetylcysteine) [41]. The recommended dose for penicillamine is 1 g/day [42]. The drug is highly efficient in reducing the excess Cu in the body and its safe long-term application has been investigated in patients. Conversely, it also shows some severe side effects such as neurological deterioration, induction in degenerative dermopathy, and cutaneous side effects [43,44]. According to a study on 179 patients with rheumatoid arthritis, after treatment with 5 mg D-Pen (control group) and with 100 mg (drug group) [45], convalescence was observed in 27% patients of the control group and 65% patients of the drug group. Some adverse reactions were also found in 34% of the control and 49% of the drug group. In the trial, the common adverse effects in the drug group were skin rashes, taste disturbances, gastrointestinal upset, and proteinuria.

Despite the side-effects, the ability of D-Pen to chelate Cu has also been investigated as an antiangiogenic property [46]. Brem et al. demonstrated that D-pen was able to remove excess serum Cu levels and reduced it to <50 μg/dL after two months of oral administration [47]. Alireza et al. demonstrated that penicillamine and low Cu diet reduces the serum Cu levels and serum Vascular Endothelial Growth Factor in the patients that underwent stereotactic radiosurgery for recurrent glioblastoma multiforme [48]. D-pen has been found to inhibit human endothelial cell proliferation in vitro and neovascularization in vivo [49]. Daizo et al. investigated a suppression in the growth of 9L gliosarcoma tumors implanted in rats when treated with D-Pen [47]. It has been studied that in the process of Cu chelation by D-pen Cu(II) reduces to its Cu(I) form which leads to the generation of hydrogen peroxide (H_2_O_2_) and other ROS [40,50]. It has been studied that D-Pen in combination with Cu promotes cell death in endothelial and lymphocyte cells due to the generation of ROS [30]. Starkebaum et al. concluded that D-Pen in the presence of Cu oxidizes to form H_2_O_2_ along with the superoxide anion (O_2_^−^) leading to the inhibition of lymphocyte mitogenesis [51]. They hypothesized that D-pen initially reduces Cu(II) to Cu(I) probably due to Cu chelation. The reduced Cu, Cu(I) then induces a reduction of oxygen and produces O_2_^−^, leading to the production of H_2_O_2_ [51]. Another report by Gupte et al. supported the possibility of the formation of ROS in human leukemia and breast cancer cells [52]. They reported that at low concentration D-Pen in the presence of Cu produces concentration-dependent H_2_O_2_ mediated cytotoxicity in cancer cells. Hence, D-pen could be employed as an anti-cancer agent owing to its dual behavior of exhibiting anti-angiogenic properties due to Cu chelation and cytotoxic properties due to the generation of ROS. However, some physicochemical properties of D-pen hinder its in-vivo anticancer activity because of limited intracellular delivery; for instance, high hydrophilicity, rapid elimination and metal catalyzed oxidation (due to which it forms an inactive D-pen disulfide or mixed disulfides) [53].

#### 2.3.2. Tetrathiomolybdate

Tetrathiomolybdate (TM) is a highly potent Cu chelating agent. Structurally it has an atom of molybdenum with four sulfur substitutions and a bidentate ligand. TM possesses dual mechanisms of action: (a) when given with meals it forms a tripartite complex of TM, Cu, and food protein; (b) in the dosage without meals, it is absorbed into the blood and forms a tripartite complex with albumin and the freely available serum Cu [28,54]. Both forms of complexed Cu are unavailable for cellular uptake. Due to its efficiency to chelate Cu and excellent safety profile, it was first used in patients with Wilson’s disease. Several clinical studies have demonstrated efficacy in treating Wilson’s disease, especially as a first-line treatment for neurologically affected patients [55,56]. The role of Cu metabolism in angiogenesis has led to the testing of TM on angiogenesis and, consequently, on carcinogenesis. TM has been shown to cause the inhibition of nuclear factor kappa B (NF-κB), a master switch for transcription of many cytokines which results in anti-angiogenesis [57]. Several studies suggest that TM suppresses tumor growth and angiogenesis [58,59]. Brewer et al. carried out a Phase I clinical trial in 18 patients with metastatic cancer and reported the reduction of ceruloplasmin, a cuproenzyme in charge of carrying Cu in the blood, without any toxicity in five of six patients with stable disease [60]. Phase II clinical trials in patients with advanced kidney cancer concluded that TM could reduce Cu levels. TM was found to be well tolerated, although its anti-cancer activity was limited to the stabilization of disease for a median of 34.5 weeks [61]. Another phase II trial of TM was performed on the patients after surgery for malignant mesothelioma [62]. This trial concluded that TM has antiangiogenic effects in malignant pleural mesothelioma patients after surgery with minimal toxicity.

Some cancer cells overexpress many receptors and proteins in their membrane and other organelles. One of them is the hypoxia-inducible factor-1α (HIF-1α), which triggers adaptive responses during low oxygen conditions, including angiogenesis, invasion, metastasis, glycolysis, tumor survival, and proliferation [63]. Cu was shown to be a requirement for HIF-1α activation, activation of vascular endothelial growth factor (VEGF) expression in cells, and promotion of wound repair in mice. TM mediated a time and dose dependent reduction of HIF-1α protein levels in human endometrial and ovarian cancer cells after 48 h of treatment [63] due to Cu deprivation. Another study showed that TM could decrease the activity of complex IV, a Cu-dependent enzyme, in the mitochondria (cytochrome *c* oxidase). In human endometrial and ovarian cancer cells, TM was the most efficient chelator to exhibit this effect [63].

#### 2.3.3. Trientine

Trien is sold under the name of Syprine, which is commonly used to treat Wilson’s disease. It is a tetradentate ligand and chemically known as triethylene tetramine dihydrochloride and was developed as an alternative drug for penicillamine intolerant patients. Trien works primarily by promoting the urinary excretion of Cu from the body. Trien was found to produce neurological worsening at the beginning of treatment but appears much less common than that with penicillamine. Trien has been shown to induce apoptosis in murine fibrosarcoma cells in vitro and in vivo [64]. Moriguchi et al. examined the antiangiogenic effect of Trien against hepatocellular carcinoma by focusing on the relationship between Cu and interleukin-8 (IL-8), which is a potent angiogenic factor produced by hepatoma cells [65]. Moriguchi et al. concluded Trien efficiently chelates Cu and prevents it from functioning as a cofactor for angiogenesis, which resulted in reduced IL-8. Another study suggests that the Trien suppresses the development of tumors and angiogenesis in the murine hepatocellular carcinoma cells [66]. A recent in vivo study suggests that Trien significantly reduces the Cu in plasma and liver tissue, additionally with the inhibition of RFA-induced inflammatory gene expression and ROS-induced malondialdehyde production in liver [67]. Trien also causes some adverse side effects, for example, reversible sideroblastic anemia, lupus like reactions, and worsening of neurological manifestations [68].

#### 2.3.4. Bleomycin

Bleomycin (BLM) is a group of glycopeptide antibiotics produced by *Streptomyces verticillus*. It is clinically used for the treatment of squamous cell carcinoma, malignant lymphoma, testicular cancer, cervical cancer, and other cancers [69]. Bleomycin causes DNA strand scission via the formation of an intermediate metal complex which requires a metal ion such as Cu(II) or Fe(III) [70,71]. BLM exhibits strong chelation with various metals and especially with Cu(II) [72]. Metal free BLM tends to bind to the Cu(II) in 1:1 stoichiometry with the formation constant value; log K = 12.63 [73]. Cu(II)-BLM complex obtains a square-pyramidal geometry [74]. Both forms—free ligand and complex form—are excellent anti-cancer agents. Due to the strong Cu(II) chelating ability BLM can reduce Cu storage in the tissue in Wilson’s disease [73].

#### 2.3.5. Curcumin

One of the most studied phytochemical compounds in Cu-based therapeutics for cancer is curcumin [75-78]. Curcumin is a bidentate ligand and binds more efficiently with redox-active metals such as Fe and Cu than the redox-inactive zinc [79]. Curcuminoid compounds have shown an excellent behavior in antitumor activity in vivo. Ahsan et al. found that curcumin in the presence of Cu(II), selectively exhibit calf thymus and supercoiled plasmid pBR322 DNA cleavage due to the formation of ROS [75]. They hypothesized that curcumin is capable of binding to DNA but in the presence of Cu(II), it shows cleavage due to the formation of hydroxyl radical (•OH). Curcumin has shown to induce apoptosis by downregulating the transcription factor NF-κB in human multiple myeloma cells [80] According to a study curcumin exhibits enhanced cytotoxicity in the cancer cells in the presence of Cu(II) selectively over the other metal ions [81]. It has been found that curcumin in combination with Cu(II) exhibits ionophoric behavior and enhances the levels of intracellular Cu leading to the suppression of the NF-κB pathway and modification of mammalian target of rapamycin-raptor (mTOR) signaling in the cancer cells [81]. A study demonstrated that curcumin efficiently chelates the Cu(II) and prevents the tumor growth, angiogenesis, and induced apoptosis in A549 xenograft model [82].

#### 2.3.6. Ionophores

Ionophores are a class of metal-binding chelators that are lipid soluble capable of transferring metal ions across biological membranes, between intra- and extracellular compartments, most commonly into the cells, but rarely out of cells [83-85]. In this section we discuss the cases of ionophores that carry metal ions into the cells. Ionophores usually transport metal ions inside the cells and can lead to the generation of toxicity in cancer cells. There are two different approaches that describes ionophoric behavior. First, moderate metal affinity allows some ionophores to bind to the metal ions from the higher concentration areas and deliver them in the area of lower concentration. Second, a suitable acid dissociation constant value (pKa) that affects deprotonation of the compound when it enters in the cellular compartments, this event induces the release of metal ions and can regulate cytotoxic behavior [86]. If the extracellular pH is higher than the pKa of the ionophore, it will form a complex with the metal ion and transport it to the environment where the pH is lower than the pKa of the compound [31]. Some Cu ionophores are selective towards cancer cells and they exhibit activity for a broad range of cancer types [14]. Bis-(thiosemicarbazone) ligands have been studied widely for their anticancer activities [87-90] and they can chelate Cu(II) after getting deprotonated at the N atoms and form neutral complexes, with the square planar N_2_S_2_ coordination geometry [90]. Cater et al. suggest that two bis-(thiosemicarbazone) Cu complexes glyoxalbis [N4-methylthiosemicarbazonato] Cu(II) [Cu(II)(gtsm)] and diacetylbis-[N4-methylthiosemicarbazonato] Cu(II) [Cu(II)(atsm)] (ligands; Figure 2), selectively kill prostate cancer cells in vitro and in vivo [90]. They found enhanced activity of both ligands when the extracellular Cu concentration increased and found them toxic when combined with Cu. They found that the Cu(II) dissociates from Cu(II)(gtsm) leading to the increase in intracellular bioavailable Cu(II) which was proven by its mechanistic action of inhibiting proteasomal chymotrypsin-like activity, an established feature associated with the Cu ionophores that increase intracellular bioavailable Cu(II) [90]. Cu in both oxidation states 1+ and 2+ can interact with electron donor groups; thiol and amino groups located on the active site of proteasome. This interaction leads to conformational changes which causes proteasome inhibition and apoptosis in cancer cells [86].

Two other compounds clioquinol and disulfiram have been investigated for their anticancer activities and are in clinical trials [31,91]. Clioquinol (5-chloro-7-iodo-8-hydroxyquinoline, CQ), and disulfiram (DSF) (Figure 2) both exhibit anticancer activities via Cu ionophoric [90] and follow the same mechanism of the inhibition of the proteasomal system chymotrypsin-like activity and induce apoptosis in cancer cells [14,83,86,92,93]. CQ is a derivative of 8-hydroxyquinoline which was initially developed as an effective amebicide for treating diarrhea [31]. CQ is a bidentate ligand which chelates metals via its N and O donor atoms and shows preference for binding to Cu(II) and Zn(II) [94]. It has been found that CQ at the micromolar concentration range induces apoptosis via a caspase-dependent pathway in eight different human cancer cell lines [31]. Studies revealed that without exhibiting any toxicity, CQ efficiently slows down the growth of xenografted tumor in mice models [84]. Caragounis et al. found that CQ elevates the intracellular Cu levels which results in the activation of PI3K (phosphoinositide 3-kinase), MAPK, and JNK (c-Jun N-terminal kinase) in amyloid precursor protein overexpressing CHO (Chinese-hamster ovary) cells [95]. In addition, when these cells are treated with the CQ and Cu(II) they exhibit a ~85–90% reduction of secreted amyloid β-peptides, Aβ-(1–40) and Aβ-(1–42) [95,96]. A sulfur-based chelator DSF is a member of dithiocarbamates and is used to treat alcoholism (FDA approved) [97]. DSF exhibits anticancer activity by the generation of ROS, inhibition of proteasome, and induction of apoptosis [94]. Denoyer et al. studied the effect of the treatment of transgenic adenocarcinoma of mouse prostate (TRAMP) cells and normal mouse prostate epithelial cells (PrECs) with the Cu-ionophores (CuII(gtsm), disulfiram and clioquinol) and found the production of toxic levels of ROS in TRAMP cells but not in the normal cells [32]. It has been studied that supplementation of DSF with Cu dramatically enhanced the inhibition of tumor growth in a prostate cancer mouse model [98]. DSF is currently under phase II clinical trial to study its impact in combination with Cu on metastatic breast cancer.

### 2.4. Efforts to Optimize Drug Delivery and Efficacy of Cu Chelator Agents

Different systems have been designed to deliver Cu chelating agents and improve their uptake from the body minimizing possible side effects. These systems take advantage of the tumor hallmarks. The enhanced vascular permeability of tumor tissue is a consequence of rapid tumor growth. Angiogenesis becomes deficient and produces blood vessels with large pores between the endothelial cells. In addition, the lymphatic drainage becomes inefficient due to the absence or malfunction of lymphatic vessels in tumors. These processes lead to the phenomenon of the enhanced permeation and retention (EPR) effect [99], which results in nanosized species penetrating the tumor vasculature and being retained for extended periods of time. Drug delivery strategies try to take advantage of this phenomenon by using nanoparticle carrier formulations of drugs. Optimal nanoparticle size range between 10 to 100 nm. The minimum size is to avoid secretion by the kidney and the larger size is to prevent phagocytic clearance by the reticuloendothelial system (RES) [100]. As drug-delivery agents, nano-carriers are capable of targeting cancer cells with enormous specificity and sensitivity especially if conjugated with ligands whose receptors are overexpressed in cancer cells versus healthy cells. If designed with a response system sensitive to the intracellular environment, they can release drugs in a regulated manner [100]. These structural fine tuning protect healthy cells from potentially toxic agents, prevent premature drug degradation, and control drug distribution over the body.

**Figure 4.**
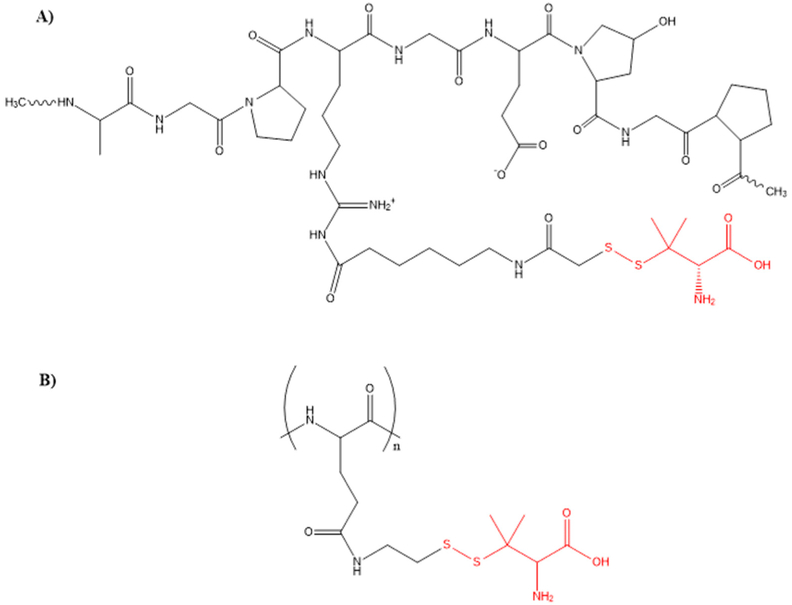
Proposed D-pen conjugates as drug delivery systems for D-pen. (**A**) Gelatin-D-pen conjugate. (**B**) Poly-l-glutamic acid (PGA)-D-pen conjugate. Reprinted with permission from *Bioconjugate Chemistry*, **19**, 1382–1388. Copyright 2008 American Chemical Society [53].

**Figure 5.**
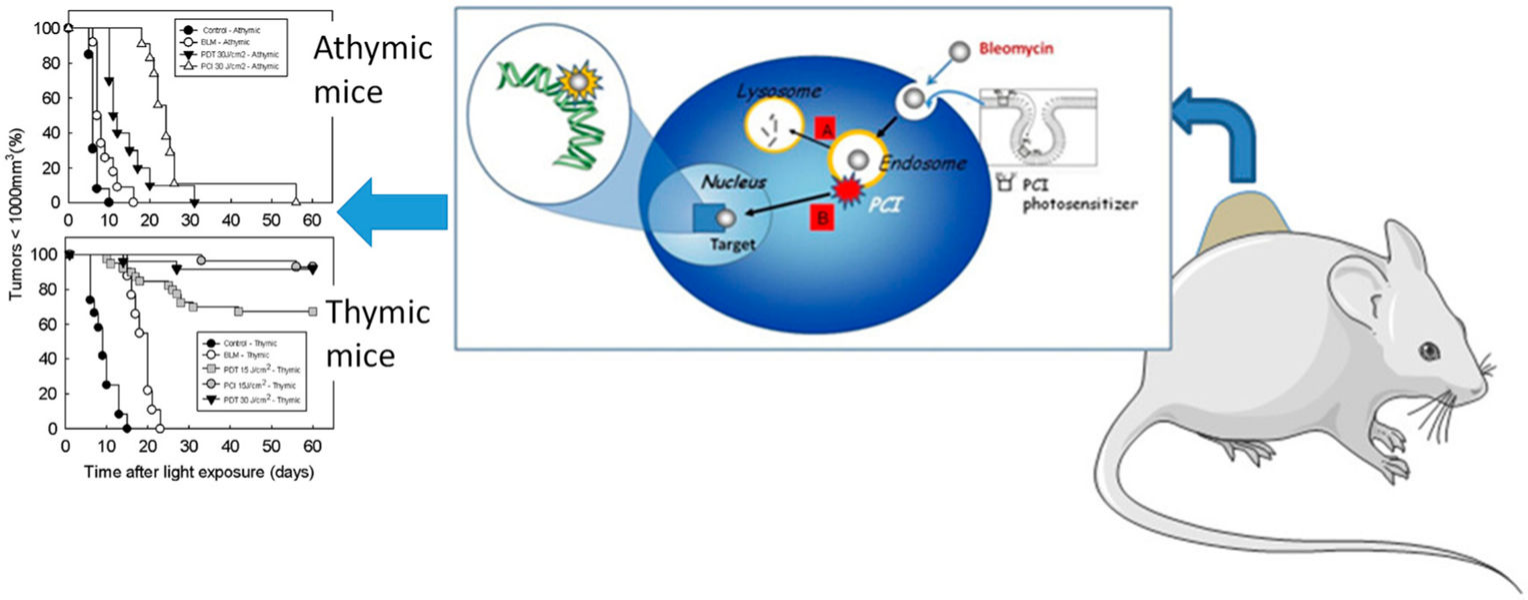
Proposed mechanism for photochemical internalization (PCI) stimulating the bleomycin release out of endosomes bringing a local therapeutic effect. Reprinted with permission from *Journal of Controlled Release*, 268, 120–127. Copyright 2017 Elsevier [102].

Efforts to improve the intracellular delivery of D-pen have been examined, taking into consideration the importance of the thiol group and the transport of high concentrations to cancer cells. This Cu chelating agent shows some disadvantages due to its high hydrophilicity. It is easy to oxidize to D-pen disulfide in vivo and it is rapidly removed from the blood [53]. One of the approaches for delivery of this drug has been the use of soluble macromolecules, such as peptides, protein, or polymers. Gupte and co-workers propose the synthesis of gelatin-D-pen conjugate with a reversible disulfide bond, resulting in the complete release of D-pen after 4 h in the presence of 1 mM glutathione at pH 7.4. Results showed cellular uptake and cytotoxicity in the HL-60 leukemia cell line [53]. Nevertheless, the system showed low efficacy. More recent methods have been reported, including the PGA-D-pen conjugate. Poly-l-glutamic acid (PGA) have the advantage of being a biocompatible and biodegradable polymer [101]. This arrangement increases the cellular uptake in vitro and the survival of mice in in vivo studies [101]. The chemical structure proposed for the gelatin-D-pen and PGA-D-pen conjugate is shown in Figure 4. Different strategies have been used to improve the cellular uptake of BLM. Norum et al. investigated the use of photodynamic therapy (PDT) and photochemical internalization (PCI) of BLM in CT26. CL25 mouse colon carcinoma cancer cells (Figure 5) [102]. PDT is a treatment well established for cancer in which a photosensitizer is used excited at a specific wavelength to produce reactive oxygen species to kill cancer cells. PCI is a recent therapy delivery technique that allows the release of macromolecules from the endosomes or lysosomes to the cytosol in the desire cancer cell. The in vivo studies implicated the use of athymic and thymic mice, in the presence and absence of T cells respectively. Delayed tumor growth in athymic mice was observed in both PCI and PDT of BLM but curative effects were not observed with the selected light dose used for PDT and PCI treatments. However, the results in thymic mice showed 90% and 70% of curative effects using PCI and PDT respectively, suggesting the importance of T cells and the immune system in general inducing cancer cells death [5,6,102].

**Figure 6.**
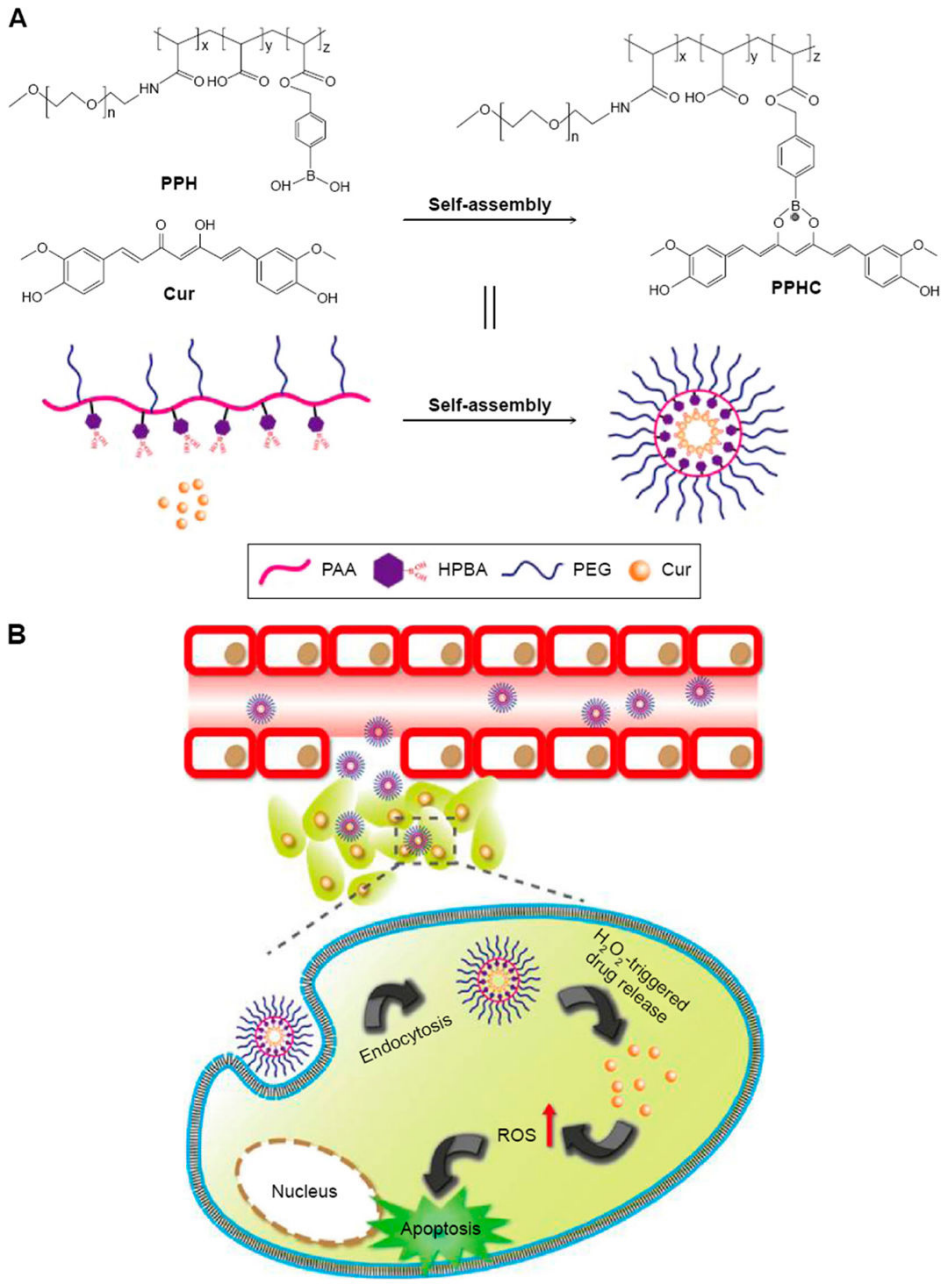
Schematic depiction of the preparation and intracellular delivery of reactive oxygen species (ROS)-sensitive responsive nanoparticle (PPHC) nanoparticles. (**A**) Preparation of ROS-responsive PPHC nanoparticles showing coordination between boronic acid and Cur. (**B**) Graphical representation of intracellular ROS-triggered drug delivery and cell apoptosis induced by amplified ROS signals. Abbreviations: PPH, 4-(hydroxymethyl) phenylboronic acid-modified PEG-grafted poly (acrylic acid) polymer; Cur, curcumin; PAA, poly(acrylic acid); HPBA, 4-(hydroxymethyl) phenylboronic acid; PEG, poly(ethylene glycol). Reprinted with permission from *International Journal of Nanomedicine*, **12**, 855. Copyright 2017 Dove Press [103].

Delivery systems have been developed to overcome the low solubility and poor bioavailability of curcumin [103]. Luo and co-workers designed curcumin-conjugated nanoparticles for delivery into A549 lung cancer cell line. Curcumin was conjugated with 1,4-(hydroxymethyl) phenylboronic acid (HPBA)-modified poly(ethylene glycol)-grafted poly(acrylic acid)polymer (PPH) in an effort to obtain a curcumin-coordinated ROS-responsive nanoparticle (PPHC) (Figure 6). Uniform spherical particles were obtained with a particle size around 163.8 nm that in the presence of H_2_O_2_ is able to release curcumin. Cellular studies demonstrated the efficient release of curcumin into A549 cells [103].

Due to the high affinity that sulfur exhibits for Cu(I) ions and the efficiency of nanomedicine, sulfur nanoparticles have been used as Cu chelators in melanoma and breast cancer cells [104]. It has been shown that Poly ethylene glycol (PEG) Sulfur nanoparticles (nano-S) are efficient as Cu chelators and selective to Cu enriched cancer cells. Cu depletion inhibited cell growth and the MEK/ERK proliferation pathway in A375 and MCF-7 cancer cells resulting in mitotic arrest [104].

In addition to efforts to improve the specificity of Cu chelators for cancer cells, other studies have been devoted to examining their synergism with other anticancer treatments. Cisplatin is used to treat different types of cancer diseases [105]. One of the issues with cisplatin is that it needs to be in high concentrations to be efficient and it could damage other cells and tissues at high dosages, while leaving the target drug resistant [105]. It has been shown that cisplatin-resistant mutants express the Cu transporter Ctr1 as a major mediator for cisplatin uptake in cells which gives platinum-based therapy higher efficacy [105]. The analysis of Cu exporters, ATP7A and ATP7B, confirmed that Ctr1 is the major determinant for cisplatin accumulation in yeast and mice cells [105]. But recent studies suggest that the transporters involved in the cellular accumulation of cisplatin are classified as; (i) Cu transporters: Ctr1, Ctr2, ATP7A, ATP7B; (ii) ABC transporter: ATOX1; (iii) organic cation transporter: OCT1; (iv) multidrug and toxin extrusion family members: MATE; (v) volume sensitive, the volume-regulated anion channel proteins: VRAC (LRRC8A/D-containing) [106]. Leucine-rich repeat-containing protein 8 (LRRC8) encoded by genes LRRC8A/D is the subunit of heteromer protein VRAC [107,108]. Cellular accumulation determination of Pt-based drugs in the cells having various LRRC8 genotypes of VRAC proteins revealed that 50–70% of long-term cisplatin uptake depends on LRRC8A and LRRC8D [109]. Studies with mice models of HPV16-induced cervical carcinoma demonstrated that the Cu chelator TM has a synergistic antitumor effect when combined with cisplatin [105]. The combination of both treatments shows an increase of cisplatin uptake into cancer cells compared to healthy cells which have a higher demand for Cu for proliferation and survival. Cancer cells overexpress Cu transporter Ctr1 which gives certain selectivity to the treatments mentioned before. The mechanism of Ctr1 is not clear but it is proposed that Cu starvation enhances cisplatin transport by changing the conformation of the Cu transporter allowing more cisplatin to enter cells and tissues. Also, TM serves as an anti-angiogenic agent and increases the efficiency of cisplatin delivery which indicates that combining both drugs will improve drug delivery and efficacy to treat different cancer diseases. A similar synergism was observed between cisplatin and the nano-S Cu chelator [104].

In conclusion, understanding Cu biochemistry especially in how Cu contributes to cancer development is useful in developing chelators as anticancer therapeutics. A number of strategies are being undertaken to improve the efficacy and uptake of Cu chelator agents into the body including formulations consisting of nanoparticles and polymers. Techniques requiring the use of light as in PDT and PCI have also demonstrated excellent value in these endeavors.

## 3. The Role of Fe in Cancer

### 3.1. Fe Transport and Regulation

Fe is the most abundant transition metal in the human body and is a vital nutrient required for several essential cell functions [110]. The forms of Fe present in the human body are heme, iron-sulfur clusters, and non-heme Fe [111,112]. Some of their biological functions are oxygen transport, energy metabolism, electron transport, cell cycle regulation, and DNA synthesis [112,113]. Fe is essential for replication, metabolism, and cell growth due to its requirement in the active site of the rate-limiting enzyme in DNA synthesis, ribonucleotide reductase (RR) [114]. The ability of Fe to convert between the ferrous form (Fe(II)) and the ferric form (Fe(III)) is the key factor in performing these biological functions [115].

Cells strictly regulate the absorption, storage, and distribution of Fe species because an imbalance in Fe homeostasis is detrimental [111]. Fe is ingested in the body in the heme and nonheme form (Fe(II)/Fe(III)) and also in metallic form fortified as in some cereal [112]. During digestion, the Fe is converted into the Fe(II) ion form and it is in this oxidation state that it is liberated from enterocytes into the blood circulation by ferroportin 1 (Fpn1), a transmembrane protein [112]. Serum transferrin (sTf) which is the major Fe transport protein can efficiently bind with Fe in its Fe(III) form [116]. For this reason, Fe needs to be oxidized for sTf to be uptaken and thus oxidation of Fe takes place at the site of Fe export. In enterocytes, the oxidation of Fe takes place by hephaestin, which belongs to a class of enzyme called multi-copper oxidase (MCOs) [112]. MCOs comprise a small family of copper-containing enzymes that oxidize substrates followed by the reduction of molecular oxygen to water [117]. Fe in +3 oxidation state is quickly uptaken by sTf in the blood, preventing loss of the metal due to its low solubility in its +3 state at pH 7.4. Fe in the blood is nearly 100% sTf bound. There is virtually little non-transferrin bound iron (NTBI), which consists of small anionic molecules such as citrate, which are likely labile in nature [118]. In cases of an overload of iron (hemochromatosis and thalassemia), Tf can be saturated and an increase in the NTBI occurs resulting in Fe(III) binding to additional anions such as phosphates and to serum albumin (the blood protein at highest concentration) and non-specific sites in Tf [118-120].

**Figure 7.**
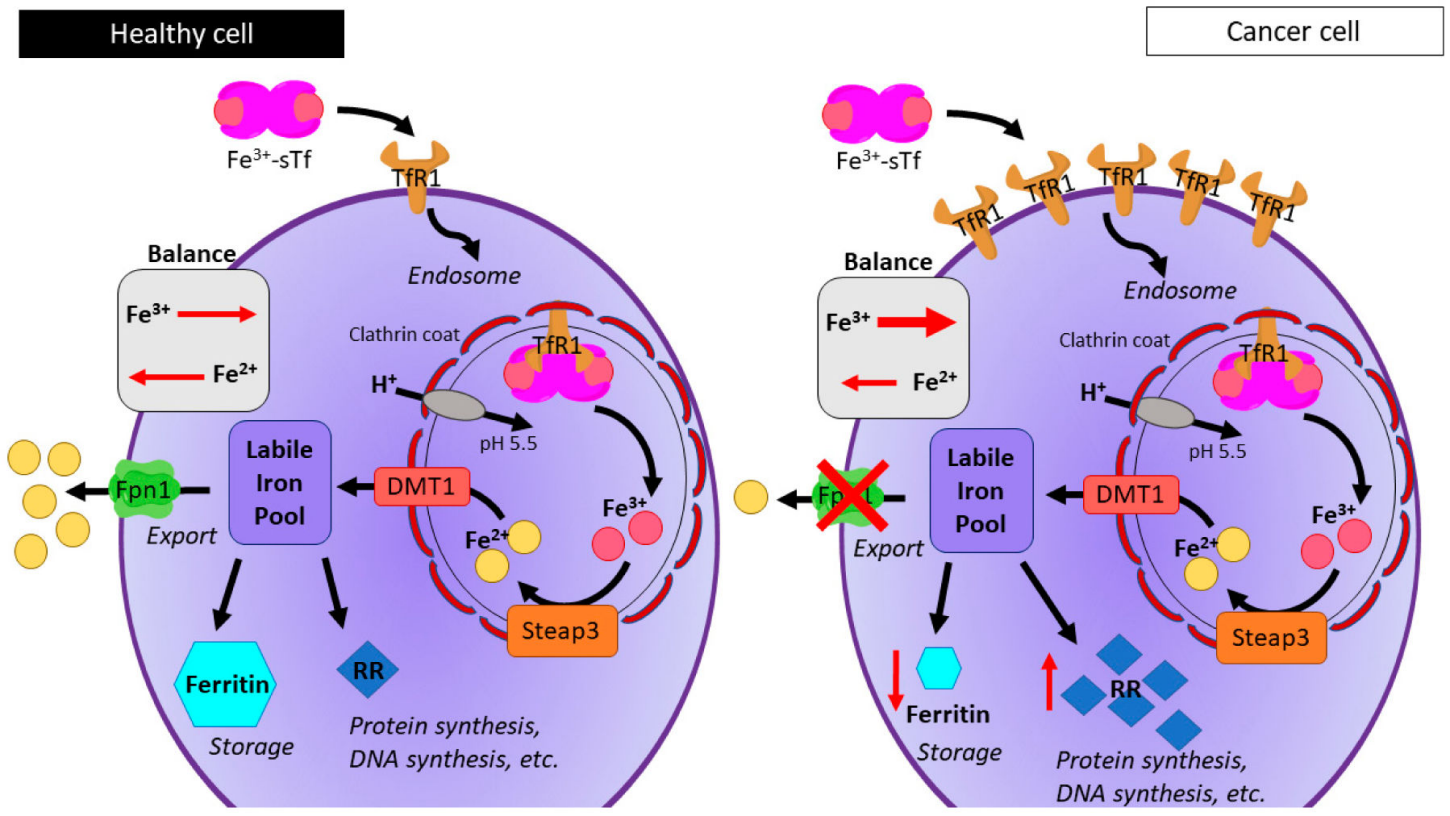
The endocytotic uptake of Fe(III) bound by sTf and trafficking and storage of Fe within the cell. In all cells, Fe(III)-bound sTf interacts with the TfR1, which then promotes the transport of Fe(III) across the cell membrane by endocytosis. A combination of pH decrease and Fe(III) reduction by STEAP3 within the endosome results in the release of Fe from sTf and transport as Fe(II) through the divalent metal transporter 1 (DMT1) into the cytosol. Fe(II) is then trafficked to several biomolecules that depend on it for activity such as ribonucleotide reductase (RR). Some of the is stored by ferritin. A portion of the Fe(II) remains part of a labile iron pool (LIP), readily accessible for use within the cell. In healthy cells there is a homeostasis between the amount of Fe that enters the cell through the transferrin receptor-1 (TfR1) and exported via ferroportin-1 (Fpn1). In cancer cells, there is a higher requirement for Fe and therefore these cells have an overexpression of TfR1 relative to healthy cells and a decreased expression of Fpn1. There is also less storage of the metal and an increased use of it. RR is overexpressed, which facilitates cancer proliferation.

Under normal conditions, sTf exclusively regulates Fe transport into cells by binding to the transferrin receptor-1 (TfR1), a cell membrane-associated a homodimeric type II glycoprotein receptor that plays a major role in the regulation of cell growth [100,112,121]. Binding to TfR1 triggers a process known as endocytosis (Figure 7) and the subsequent endosome that forms is protected by becoming coated with the clathrin protein. The TfR1 homodimer is held together by disulfide linkages and constitutively endocytosed through the canonical clathrin-mediated pathway. Once in acidified endosomes, a receptor carrying iron loaded Tf undergoes a structural rearrangement that promotes the release of iron by Tf and then the iron-free Tf molecule is recycled back to the cell surface [121]. A combination of acidification and the reduction by STEAP3 results in the dissociation of the metal from sTf in the +2 oxidation state [122-126]. It is then released from the endosome into the cytoplasm via the divalent metal transporter 1 (DMT1). DMTs are transmembrane proteins that exist in isoforms, for instance DMT1 and DMT2 [127]. DMT1 specifically transports Fe(II) and other divalent but not all metal ions [128]. The Fe-free sTf is then returned to the membrane and released back into the blood to be recycled for further rounds of Fe cellular transport. Fe(II) ions are then trafficked to different parts of cells for a variety of functions [129] and also to the protein ferritin for storage. A small but important pool of labile Fe(II) remains in the cytoplasm for later insertion into biomolecules that depend on Fe for activity [130,131].

### 3.2. The Role of Fe in Cancer and Its Progression

Improperly sequestered Fe(II) or the excessive build-up of the labile Fe pool catalyzes the overproduction of ROS through Fenton chemistry, which can lead to cell death and disease [115]. Fenton in 1894, conducted an experiment that identified the role of Fe in the production of •OH, a reaction called the Fenton reaction [132]. O_2_^−^ and H_2_O_2_ damage the iron-sulfur clusters of dehydratases amongst other protein, releasing Fe ions and raising the levels of the labile Fe pool [133,134]. Any Fe(III) that is released from the proteins is quickly converted into the Fe(II) form because of the presence of O_2_^−^ and the variety of reducing agents such as glutathione, NADH, and ascorbic acid in addition to redox active enzymes within the cell that create a reducing environment [134]. Fe(II) is then able to interact with oxygen leading to the production of H_2_O_2_ to initiate the Fenton reaction and yielding the •OH radical [135]. This overall process is called the Haber–Weiss reaction [136]. In cells, O_2_^−^ is chemically incapable of directly damaging DNA but serves as a reducer for Fe that is additively bound to DNA [134]. This reaction not only damages lipids and proteins, but also causes oxidative damage to DNA, including DNA base modifications and DNA strand breaks which can be mutagenic [111,114]. Mutagenesis by the excessive production of ROS could contribute to the initiation of cancer, in addition to being important in the promotion and progression phases [129]. Consequently, elevated levels of Fe have been identified as a risk factor for the development of cancer [137,138]. Fe-induced malignant tumors were first reported in 1959 by repeated intramuscular injection of Fe dextran complex in rats [139]. Many years later, several studies have reported observations of Fenton reactions in diverse types of cancer [114]. For these reasons, Fe is correlated with carcinogenesis and cancer progression.

The proliferation of tumor cells requires sustenance in the form of nutrients and oxygen [140]. Therefore tumor cells require more Fe which results in an increased expression of the transferrin 1 receptor (TfR1) for the endocytotic uptake of Fe(III) (Figure 7) [100,141]. Numerous studies showed that cancer cells overexpress TfR1 when compared to normal cells [131,142-144]. This is attributed to the increased need for Fe as a cofactor of RR, also overexpressed, involved in DNA synthesis of rapidly dividing cells [145]. Brookes et al. reported that the progression of colorectal cancer is associated with increased expression of Fe import proteins, such as DMT1 and TfR1, and decreased expression of the Fe export protein, Fpn1, which lend to an increased labile iron pool (LIP) [146]. Pinnix et al. demonstrated that Fpn1 abundance was reduced in aggressive some breast cancer cell lines when compared to healthy cells [147]. In addition, the metalloreductase, STEAP3, is overexpressed in cancer cells, which would increase the rate endosomal Fe(III) reduction to Fe(II) and subsequent release from the endosome [114]. In the process of metastasis, some studies have identified that tumor cells express Fe-containing matrix metalloproteinases that degrade the extracellular matrix and assist in the invasion of cancer cells [148,149]. As described previously, Cu contributes to the overexpression of matrix metalloproteinases, which shows an interplay between the excessive levels of Cu and Fe in cancer progression.

In summary, many studies have demonstrated that an increase in the entry of Fe, a fall in its elimination, and an interruption in its storage inside the cell result in an accumulation of Fe that leads to an increase in the risk of cancer [114]. Fe inside the cell is available for DNA synthesis, cell proliferation, or the formation of ROS [135]. Due to the ability of Fe within the cell to catalyze ROS formation especially when exceeding homeostatic levels, it promotes many aspects of tumor development and progression [150].

### 3.3. Fe Chelators in Cancer Treatment

**Figure 8.**
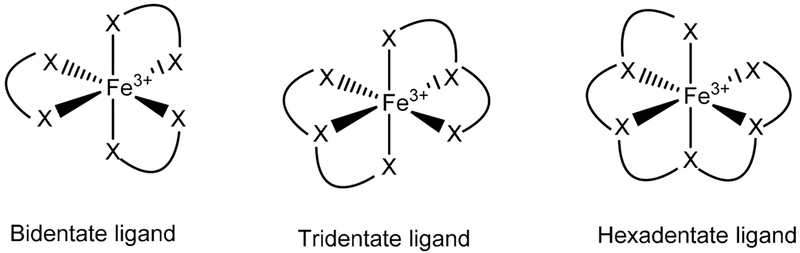
General Fe(II) and Fe(III) coordination by bidentate, tridentate, and hexadentate chelators to satisfy 6-coordinate structures.

**Figure 9.**
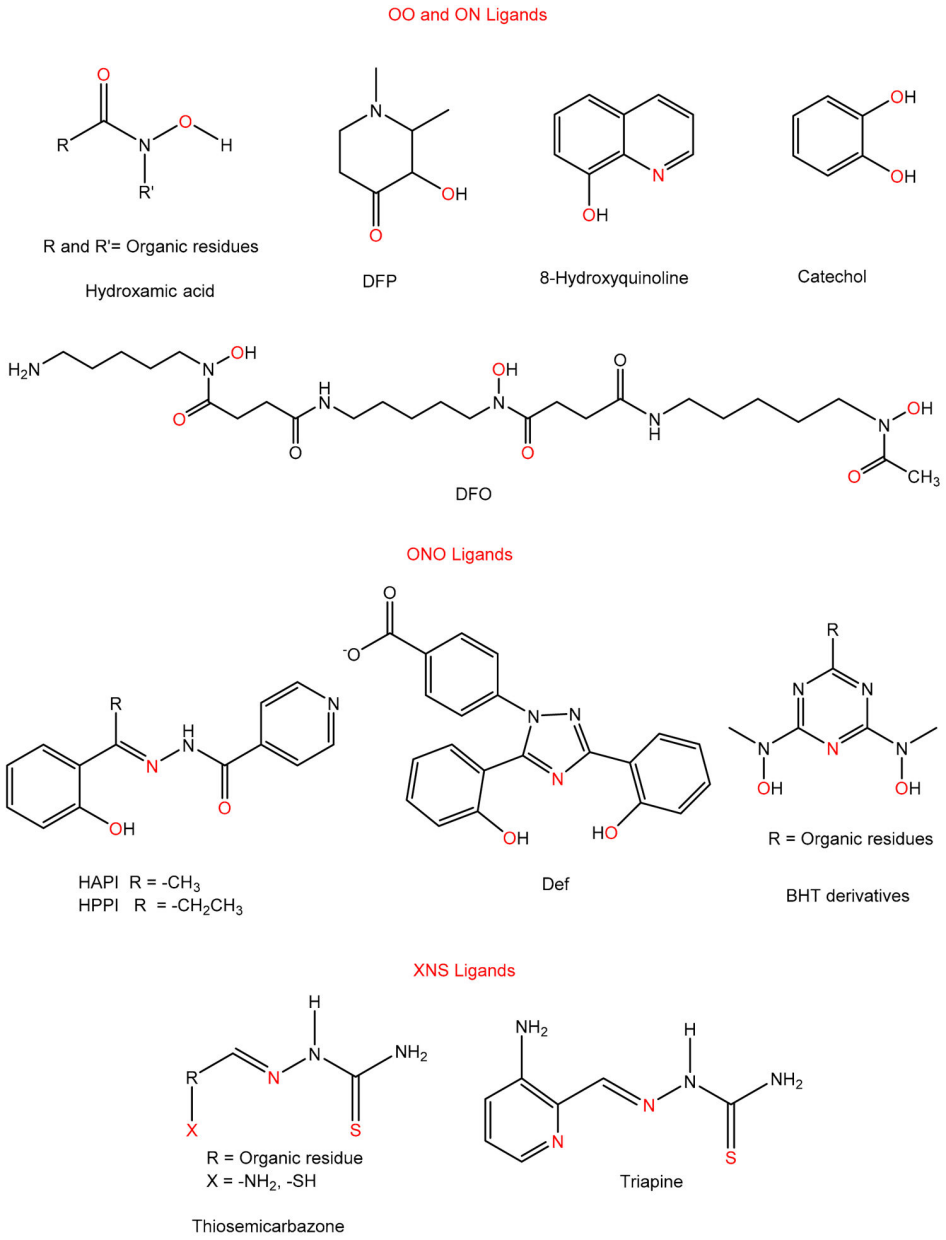
The structure of selected Fe chelators for potential anticancer application divided into the OO, ON, ONO, and XNS family of ligands. DFP: Deferiprone; DFO: Deferoxamine; BHT: 2,6-bis[hydroxy-(methyl)amino]-1,3,5-triazine; HAPI: (*E*)-*N′*-[1-(2-hydroxyphenyl)ethylidene]-isonicotinoylhydrazide; HPPI: (*E*)-*N′*-[1-(2-hydroxyphenyl)propylidene]isonicotinoylhydrazide.

Several Fe chelators that were originally developed for the treatment of Fe overload in different diseases [151] were later found to reduce tumor growth by different possible processes that regulate the cell cycle, angiogenesis, or the suppression of metastases [8,152,153]. Fe in the +2 and +3 oxidation states forms coordination compounds that are typically six-coordinate. For this reason, Fe chelators for clinical use have been designed in bidentate, tridentate, or hexadentate modalities to satisfy this “preferred” coordination number (Figure 8). Fe(II) is an intermediate Lewis Acid whereas Fe(III) is a hard Lewis Acid and both Fe ions can bind to oxygen, nitrogen, and depending on the denticity even sulfur donor atoms [34]. The Fe(II) and Fe(III) chelators considered for anticancer application can be divided into the OO, ON, ONO, and XNS family of ligands (Figure 9). In recent years there has been an exhaustive collection of review articles on Fe chelators [141,154-156]. To avoid repetition, we herein highlight a few features about prominent Fe chelators that have the capacity to bind extracellular Fe(III) and intracellular Fe(II)/Fe(III). We explore how their role in suppressing cancer can provide insight into the next generation of anticancer chemotherapies.

#### 3.3.1. OO and ON Ligands

Deferoxamine (DFO) (Figure 9) is a bacterial siderophore [157] that has the capacity to bind Fe in a hexadentate fashion forming a 1:1 metal:ligand complex. It is a member of the hydroxamate class of ligands. DFO is clinically used for the treatment of Fe overload and was the first Fe chelator to be tested for anticancer applications [158]. It inhibits melanoma and hepatoma cell growth both in vivo and in vitro by blocking their proliferation in the S phase of the cell cycle [159]. The mechanism of DFO in hepatocellular carcinoma cell lines (HCC) was investigated by monitoring Tf-^59^Fe uptake in the cells [160]. Compared with the controls, the cells treated with DFO showed less uptake of ^59^Fe, which indicates that the compound chelates the iron extracellularly. DFO was effective in the treatment of leukemia and neuroblastoma during preliminary clinical trials [159]. Nonetheless, DFO has a short plasma half-life, and is markedly hydrophilic, which makes it ineffective when administered orally. Deferiprone (DFP) (Figure 9) is a bidentate ligand that makes 3:1 ligand:Fe(III) complexes at physiological pH with Fe(III) [161]. DFP is approved in the United States for the treatment of thalassemia but also shows antiproliferation activity. It has been found that DFP acts as a pro-oxidant or protective anti-oxidant and can reduce Fe concentration in cells as much as DFO.

Studies have been conducted to explore modifications of Fechelators to incorporate functional groups with the hopes of inducing a synergistic antiproliferative effect. To that end Qiao et al. synthesized endoperoxide conjugates of derivatives of the Fe chelators hydroxamic acids, catechols, and 8-hydroxylquinolines (Figure 9) [162]. The endoperoxide is proposed to become activated in the presence of cellular Fe. Following treatment with the conjugates, the cell viability of five human cancer cells HL-60 (leukemia), A549 (lung), SW480 (colon), and SMMC7721 (liver), and the non-cancer hepatocellular cell (HL7702) was measured [162]. The 8-hydroxylquinoline conjugates exhibited the highest antiproliferative effect with IC_50_ values that were generally less than 10 μM. These conjugates displayed a greater than three selectivity index, suggesting their selectivity towards attacking cancer cells. The mechanism of action of the conjugates was owed, in part, of inducing apoptosis and to the higher stability of the Fe compounds of these conjugates relative to the other chelators.

#### 3.3.2. ONO Ligands

Deferasirox (Def) (Figure 9) is a tridentate chelator that forms a 1:2 Fe(III):ligand complex at pH 7.4 [163]. It is commercially used as an oral drug for the treatment of Feoverload. Def is currently being tested against several cancer cell lines [164-167] because it has the capacity to induce DNA fragmentation, inhibit DNA synthesis, deplete Fe, trigger caspase-related apoptosis, amongst other activities.

The (*E*)-*N′*-[1-(2-hydroxyphenyl)ethylidene]isonicotinoylhydrazide (HAPI) and (*E*)-*N′*-[1-(2-hydroxyphenyl)propylidene]isonicotinoylhydrazide (HPPI) Fe semicarbazone chelators (Figure 9) also demonstrate antiproliferative properties. Vávrová et al. prepared derivatives of these chelators by modifying the hydrazide component of the molecules [168]. The derivatives were evaluated for their Fe-chelating abilities, anti/pro-oxidative properties, capacities to prevent Fe(III) uptake from transferrin, amongst other properties in MCF-7 (human breast cancer) and HL-60 (leukemia) cell lines. To compare intracellular Fe chelation, the calcein assay was used [169,170]. The HAPI analogues with a nitrogen heterocycle in the hydrazide part of the molecules showed similar or better Fe chelating activities than the standard chelators and greatly improved hydrolytic stability in plasma. It was also demonstrated that some of the derivatives with increased lipophilicity retained comparable antiproliferative activity as the unmodified HAPI and HPPI. The same compounds also showed high selectivity ratios, specifically the biphenyl-containing ligands.

The 2,6-bis[hydroxy-(methyl)amino]-1,3,5-triazine (BHT) family of tridentate chelators (Figure 9) [171] consists of the following properties: (i) strong binding of Fe(III) along with high Fe(III)/Fe(II) selectivity resulting in very low redox potential of the formed Fe(III) complex which precludes uncontrolled formation of reactive oxygen species in normal cells; (ii) rigidity of the ligand, resulting in size-selectivity towards Fe(III) binding; and (iii) balanced hydrophobicity of the ligand, allowing it to be soluble in aqueous media and membrane permeable. The in vitro antiproliferative behavior of BHT analogues against two human cancer cell lines, MDA-MB-231 (breast cancer), and MiaPaCa (pancreatic cancer) was assessed [171]. The results suggested that the substitution on the nitrogen or oxygen atoms of the hydroxyamino groups is essential for modulating the cytotoxic activity of the BHT analogues and improving selectivity toward cancer cells versus normal cells.

#### 3.3.3. XNS Ligands

Like the semicarbazones, thiosemicarbazones (TSC) (Figure 9) are effective Fe(II) and Fe(III) chelators. TSC are versatile ligands that exhibit wide pharmacological properties for instance, antineoplastic, antibacterial, antiviral, and antifungal activity [33,172]. Their antineoplastic mechanism of action includes the activities for instance; (a) inhibition of cellular iron uptake from transferrin, (b) mobilization of iron from cells, (c) inhibition of RR, and (d) the formation of ROS [173,174]. One of the best studied TSC compounds is Triapine. Triapine (3-AP) was initially developed as a potent RR inhibitor, which uses radical-based catalysis to form deoxyribonucleotides. Its exact mechanism of inhibition is still being researched, but it is believed to be caused by ROS generation, specifically by the formation of the 3-AP Fe(II) complex, and/or depletion of cellular iron pools [8]. Triapine has undergone Phase I and II clinical trials for cancer treatment [153], because of the anti-proliferative activity against a number of cancers [172], including L1210 leukemia cells both in vitro and in vivo [175,176].

The use of iron chelators in the treatment of cancer does not require blockage or depletion of the metal from the cell but rather attenuating the toxicity or functionality of the metal to then detrimentally alter the intracellular processes of tumors. Koppenol et al. found that Fe complexes with some clinically used chelators such as desferrioxamine, deferiprone, and deferasirox exhibit a large negative electrode potential, which prevents the toxicity that is generated by Fe redox cycling from excessive labile Fe [177]. Some iron chelators interfere with the cell cycle and/or inhibit the reproduction of DNA, angiogenesis, cellular growth, amongst other molecular mechanisms that depend on Fe. Studies with iron chelators of clinical interest have revealed strategies that could prevent the onset of certain cancers and provide new molecular targets that are Fe dependent.

### 3.4. Efforts to Optimize Drug Delivery and Efficacy of Fe Chelator Agents

Fe chelators can prevent Fe acquisition or utilization by cancer cells. This results in a pronounced anti-proliferative effect due to the inactivation of the RR [178]. This discovery propitiated researchers to start developing novel Fe chelators, looking to exploit their strong Fe-binding capabilities for anti-proliferative cancer therapy. Many Fe-chelating compounds have been developed, and a few are even FDA-approved, such as DFO, DFP, and Def [179]. However, these compounds present limitations due to their acute toxicity and short plasma life [180]. For example, DFO is limited by its rapid excretion, metabolic breakdown, and low cellular uptake [159,180]. The demand exists for novel methods that can reduce the toxicity of these Fe chelators and enhance the bioavailability of these compounds in target tissues. We will discuss some innovative drug delivery methods, prochelation, and synergistic treatment approaches to improve the cellular specificity and/or efficacy of Fe chelators.

#### 3.4.1. Nano-Approaches for Delivering Fe Chelators

Nanoparticles based on poly(lactic-*co*-glycolic acid) (PLGA) are being widely used as delivery agents because of their biocompatibility, ease of attachment of targeting molecules, and ability to fine tune the release of bound compounds [178]. Di-2-pyridylketone-4,4-dimethyl-3-thiosemicarbazone (Dp44mT) is an Fe chelator compound that has previously shown great anti-proliferative characteristics in several cancers including breast cancer and melanoma [178]. A PLGA-NP was successfully developed with high encapsulation efficiencies and efficient release of Dp44mT [179,180]. Cell viability assays indicate that Dp44mT in free form and PLGA-NP encapsulated were highly effective in killing U251 glioma cells, suggesting that encapsulation of this compound in PLGA NPs did not affect its activity [179]. These findings suggest that PLGA-NPs are suitable carriers for efficient encapsulation of Dp44mT, which increases the number of drug particles that actually reach the tumor. This results in a targeted delivery of this Fe chelator to malignant cells.

Abayaweera et. al. reported the synthesis, characterization, and efficacy of Fe/Fe3O4-nanoparticles co-labeled with a tumor-homing and membrane-disrupting oligopeptide and the Fe chelator Dp44mT [181]. This Fe chelator and the peptide sequence PLFAERL (D[KLAKLAKKLAKLAK])CGKRK were conjugated to the surface of the nanoparticles using dopamine anchors [181]. The peptide sequence has two important parts, one responsible for tumor targeting and the other is responsible for the disruption of mitochondrial cell walls and triggering apoptosis [182]. In this study, the activity of the nanocarrier was tested on the highly metastatic 4T1 murine breast cancer cell line and was compared with a non-cancerous murine skin fibroblast cell line (MSFs) [181]. It was observed that at an optimal ratio of 1 to 3.2 of the peptide sequence and the Fe chelator, the IC_50_ value was 2.2 times lower for the breast cancer cells than for the control cell line [181]. These were encouraging results but further studies in different compositions of nano-carriers are necessary to achieve higher efficacies and better specificity.

Polymeric micelles are self-assembly nanocarriers with core/shell structures formed by amphiphilic block copolymers [183]. The core can enclose the hydrophobic drug with a hydrophilic shell, increasing the solubility and stability [167,183]. PEG is commonly used as the shell because of its stealth property and hydrophilicity which allows micelle to avoid recognition by the mononuclear phagocyte system (MPS) [167]. MPS is a class of cells that occur in separated parts of the human body and that have in common the property of phagocytosis, whereby the cells engulf and destroy bacteria, viruses, and other foreign substances and ingest worn-out or abnormal body cells [184]. Theerasilp et al. presented the modification of the Fe-chelating drug Def (Figure 9), by conjugation of a pH-sensitive moiety encapsulated into PEG-b-PCL micelles as a drug delivery strategy for cancer chemotherapy [167]. Release studies of Def and its derivatives including methoxy (mDef) and imidazole-modified (iDef) deferasirox micelles were tested under simulated lysosomal condition (pH 6.0 and 5.5) representing different stages of endocytosis [167]. The results showed that in iDef-loaded micelles, a small change in pH environment (intracellular pH gradient) caused a significant effect of the drug release rate [167]. The cytotoxicity assays of the micelles loaded with Def, mDef and iDef demonstrated anti-proliferative properties suggesting that they have potential applications for cancer treatment, especially micelles loaded iDef [167]. These micelles showed cytotoxic effect against the PC-3 cell line in sub-micro molar level [167]. The IC_50_ of drug-loaded micelles was slightly higher than that of the free drug because of the prolonged release of the drug from micelles. The IC_50_ values of iDef-micelles in normal and cancer cell lines showed promising antiproliferative activity with minimal cytotoxicity to the normal cell lines [167].

#### 3.4.2. Liposomes for Delivering Fe Chelators

Traditional drug delivery systems are engineered to yield a sustained release of bioactive compounds. Liposome drug delivery systems have played a significant role in delivering significant amounts of drug to improve therapeutic outcomes [185]. Recently, the liposome formulations are targeted to reduce toxicity and increase accumulation at the target site, which has been a limitation with conventional delivery systems for Fe chelators. Most clinical applications of liposomal drug delivery are focused on targeting tissue with or without expression of target recognition molecules on lipid membranes. This mode of drug delivery lends more safety and efficacy to administration of several classes of drugs like antiviral, antimicrobial, vaccines, and gene therapeutics. Recent developments in this field have demonstrated the specific binding properties of a drug-carrying liposome to a target cell such as a tumor cell and specific molecules in the body [179]. This ability can be exploited for the efficient delivery of Fe chelators.

O’Neill et al. examined the timed-release capabilities of lysolipid-based thermosensitive liposomes (LTSL) embedded in a chitosan-based thermo-responsive hydrogel matrix (denoted Lipogel). They tested their model by using DFO (Figure 9), the chelator of choice for removal of excess stored Fe. The group loaded their chitosan hydrogels with either free DFO or LTSL-encapsulated DFO and proceeded to test the release of DFO in PBS for 10 days. Their results demonstrate a sustained and prolonged release of DFO using LTSLs embedded in a hydrogel matrix [180]. With these experiments, they proved that the entrapment of drug-loaded liposomes in an injectable hydrogel permits local liposome retention, thus providing a prolonged release in target tissues [159,180]. They also showed that release of DFO can be controlled through the use of an external stimulus [180]. In this case, stimulus consisted of a hyper-thermic pulse of 42° for a period of 1 h. Their experimental model consisted of irradiating hydrogel particles containing 100 μM LTSL-encapsulated DFO with the hyper-thermic pulse on day 2, 6, or 10 [180]. An initial burst release of drug was observed in the first 24 h due to passive DFO diffusion from the LTSLs. A second burst release was observed after application of the hydro-thermic pulse at different time points. With this, they demonstrated that their system is capable of delivering two different doses of drug at different time intervals [180].

#### 3.4.3. Prochelation

**Figure 10.**
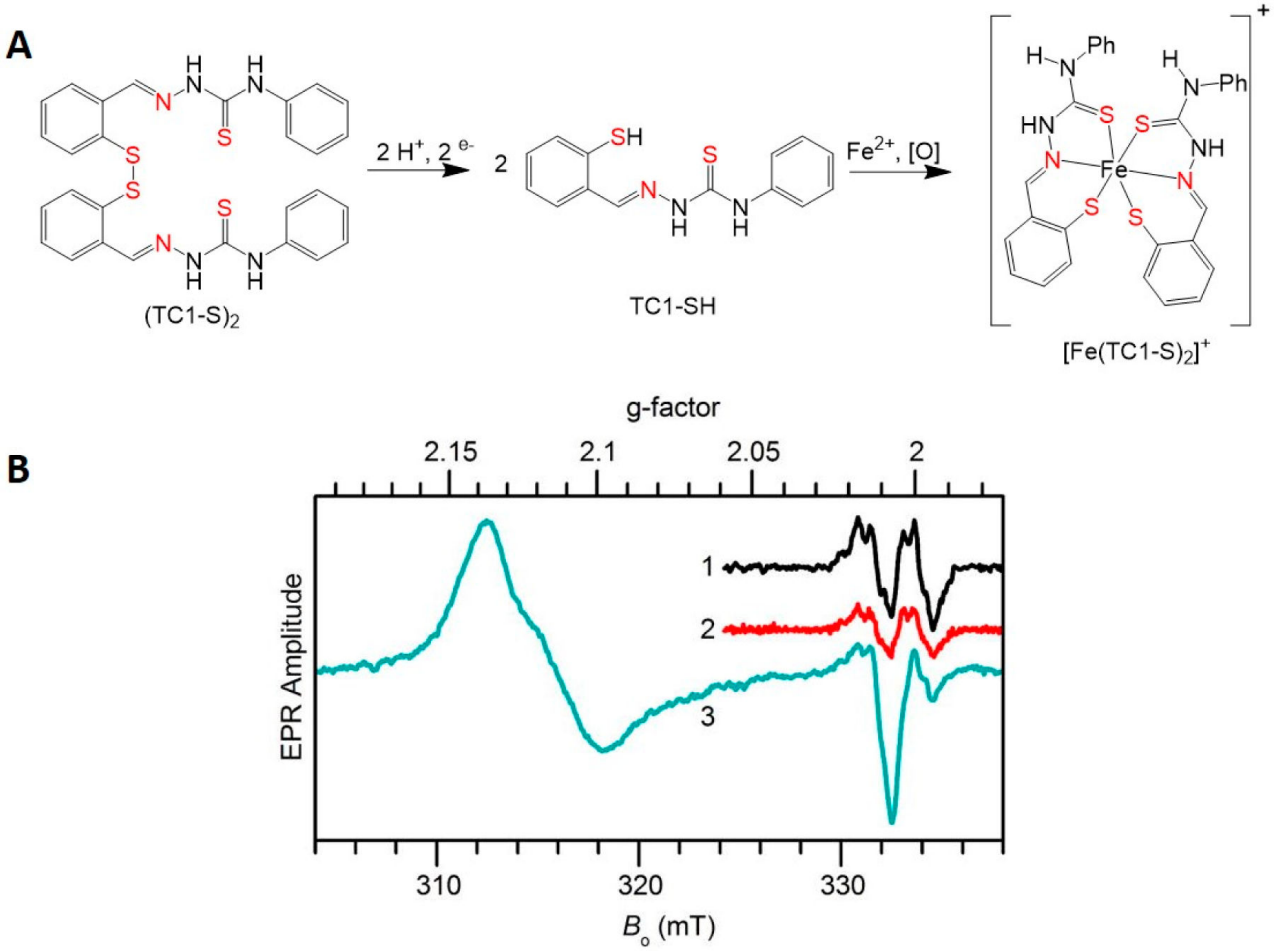
**(A)** Schematic of the reduction of the prochelator (thiosemicarbazones (TSC)-S)_2_ for the activation of an antiproliferative thiosemicarbazone chelator (TC1-SH). This chelator would bind Fe from the LIP. (**B**) Enhanced permeation and retention (EPR) spectra of whole Jurkat cells: (1) untreated cells, (2) after treatment with 50 μM DFO for 3 h, and (3) after treatment with 50 μM (TC1-S)_2_ for 1 h. Experimental conditions: microwave frequency, 9.338 GHz; microwave power, 2 mW; magnetic field modulation amplitude, 0.5 mT; temperature, 30 K. Reprinted with permission from *Metallomics,* 6, 1905–1912. Copyright 2014 Royal Society of Chemistry [187].

Prochelation strategies, in which a chelator is formed in response to a triggering event, are being developed to increase the preferential activity of metal chelators within cancer cells versus healthy cells. One such approach has been undertaken by Tomat et al. using the thiosemicarbazone tridentate scaffold (Figure 9) [186-189]. They have included a redox responsive disulfide bond switch that following reduction in the reducing environment of cells activates the formation of the chelator. This switch is particularly sensitive in cancer cells because of their higher GSH concentrations relative to normal tissue. Tomat et al. have found that the thiosemicarbazone prochelators attenuate the bioavailability of Fe within cancer cells and as a consequence inhibits the activity of the RR enzyme. This finding was demonstrated by the decrease in the g ~ 2 electron paramagnetic resonance (EPR) signal associated with the active enzyme’s tyrosyl radical and the growth of a low spin Fe(III) signal in a nearby region (Figure 10). Structural modifications to the prochelators have been made to incorporate functional groups that would allow targeting of specific tumor types [188].

#### 3.4.4. Synergistic Treatment

The clinical use of 3-AP is potentially limited by its low efficacy in some trial studies and possible toxicity related to its dosage [8]. Synergism between compounds has been an area of interest for researchers regarding the anticancer activity of TSC compounds. One way to enhance the ROS formation from TSC compounds is with PDT [190]. This would influence the cellular redox environment of the cells, leading to the induction of oxidative stress. It has been shown that mixtures of PS and TSC increases the production of singlet oxygen, provoking different types of cellular damage, including lipid peroxidation and accumulation of ROS in the mitochondria, due to the labile mitochondrial iron metabolism [191]. Another important characteristic of TSC, is the ability to upregulate the metastasis suppressor NDRG1 [173]. NDRG1 expression is induced by cellular iron depletion, thus effective iron chelators render a promising therapeutic ability through a different pathway. A more potent TSC was generated, di-2-pyridylketone-4-cyclohexyl-4-methyl-3-thiosemicarbazone (DpC), which showed complete inhibition of pancreatic tumor growth [192]. Amongst the targets that DpC affects are: (a) NDRG1; (b) p21CIPI/WAF1 a cyclin-dependent kinase inhibitor; and (c) cyclin D1, which is necessary for the cell cycle progression, by the depletion of iron [193]. Cyclin D1 is known to function as an oncogene in pancreatic cancer, usually being overexpressed in these tumors [194], hence anticancer agents that are able to reduce cyclin D1 levels are beneficial for the treatment of pancreatic cancer. In a study against pancreatic cancer drugs gemcitabine and 5-fluorouracil, DpC’s IC_50_ values were at least 4- and 2000-fold lower respectively in four pancreatic cell lines [8]. Dp44mT, a DpC analogue also showed potent anti-cancer activity against various cancer cell lines, with IC_50_ values in the nanomolar range, even against cells lacking the important tumor suppressor protein that prevents tumorigenesis p53 [174]. Moreover, it was found that Dp44mT increased Fe mobilization, resulting in the release of 38% of total cellular Fe, as well as reducing its uptake to less than 10% of the control sample [174].

In summary, there are various drug delivery methods, structural modifications, and synergistic approaches that can mitigate some of the issues with common Fe chelators. Nanoparticle-based therapeutic systems can enhance the bioavailability of the drug, plasma retention time, and produce a stronger anti-tumor effect. A targeting ligand can be attached to carriers in order to increase the selective uptake in tumors and reduce adverse side effects. Responsive systems can be designed for improved drug release in cells and for exclusive activation in cancer cells. Combining chelators with other therapeutic strategies could enhance their cytotoxicity while minimizing their general toxicity.

## 4. Transmetalation as a New Anticancer Strategy to Target Cu and Fe Chelation

A major limitation in the use of Cu and Fe chelators is that although they have high affinity for these metals and thus are termed selective for them, they still have the capacity to bind other metals and can perturb their homeostasis in the body. The ability of Cu and Fe chelators to bind other metals can be exploited in a therapeutic manner by the judicious choice of transmetalation. In this approach other metals are introduced into the cells in compound form with the Cu or Fe chelators and in the presence of the labile Cu and Fe pool, metal exchange occurs releasing the external metal. The released metal will then synergize with the chelators to enhance the antiproliferative and/or cytotoxic effect. For this approach to be successful, the metal needs to display potent cell killing capacity and also be able to form a stable compound with the chelator that only in the intracellular environment undergoes induced dissociation by the labile Cu and Fe pool at physiologically fast rates [195,196].

**Figure 11.**
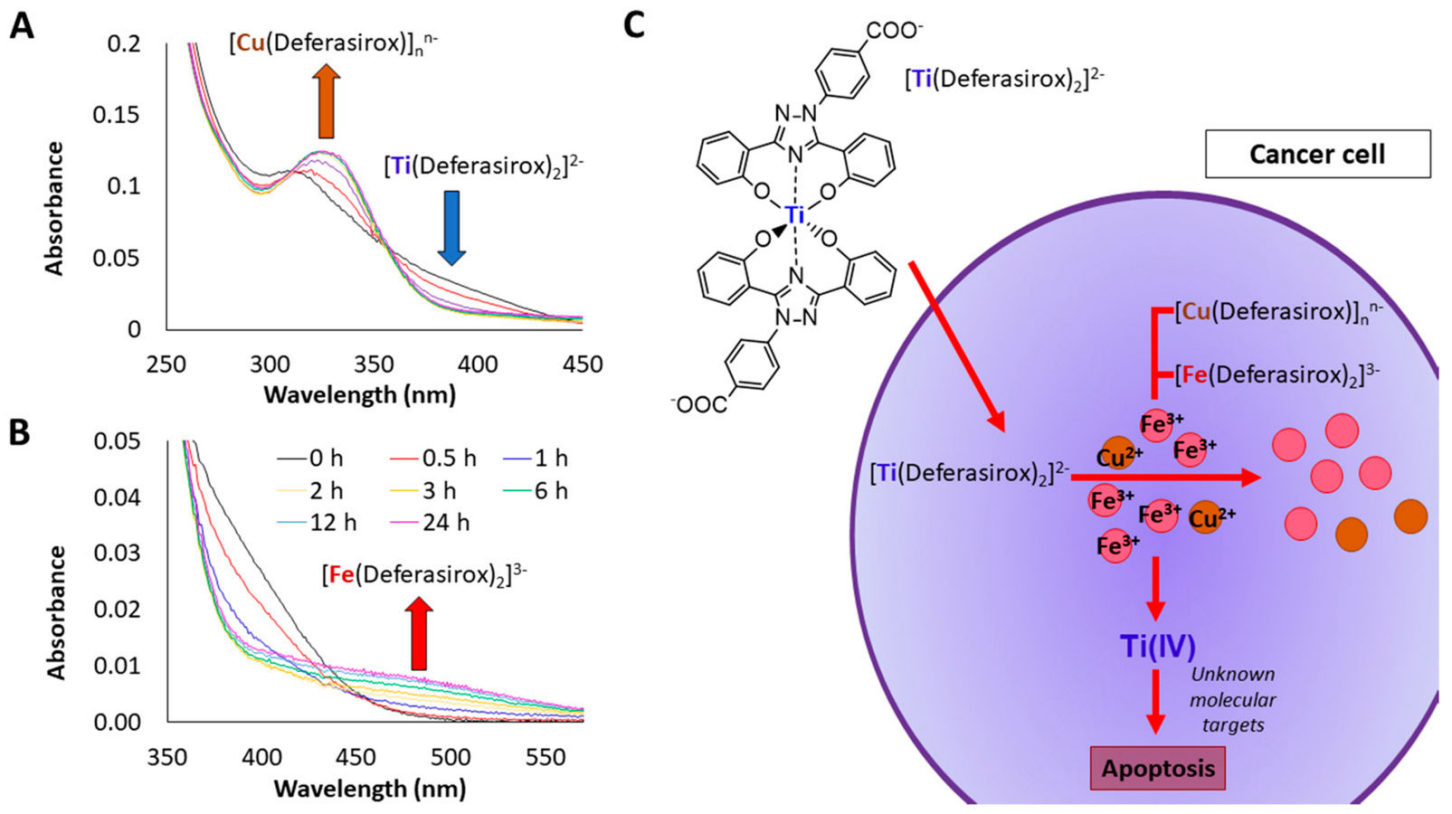
The transmetalation reaction from the simultaneous reaction between Ti(deferasirox)_2_^2−^ and labile sources of Fe(III), Cu(II), and Zn(II) monitored by UV-Vis spectroscopy. (**A**) Growth in the ligand to metal charge transfer absorbance for [Cu(Def)]*_n_^n^*^−^ is observed very rapidly. (**B**) Growth in the ligand to metal charge transfer absorbance for [Fe(Def)]_2_^3−^ is observed. (**C**) Proposed model for the intracellular transmetalation of Ti(deferasirox)_2_^2−^ by Cu(II) and Fe(III) leading to release of Ti(IV) and subsequently apoptosis.

In metal-based therapeutics, transmetalation can be a detriment because of the exchange of essential metals in the body for the metals delivered in the drugs [197,198]. For instance, platinum(II) from Pt(II)-based anticancer compounds and gold(I) from Au(I)-based Rheumatoid arthritis compounds have been shown to transmetalate with zinc(II) in undesired parts of the body and decrease the bioavailability of the metal. Pt(II) can displace Zn(II) from human serum albumin [199], one of its main blood transporters, and Au(I) can displace Zn(II) from zinc finger protein sites [200]. With careful consideration of coordination chemistry, these undesirable transmetalation events can be avoided. To this end, Tinoco et al. has sought to manipulate the chemical proximity of titanium(IV) for Fe(III) for a transmetalation anticancer strategy [201,202]. Ti(IV) mimics the coordination chemistry of Fe(III) in being able to bind the same biomolecules though not always in the same coordination modalities [203]. Ti(IV) in chelate form is typically redox inert and this property distinguishes it from Fe because many of its biological functions depend on its redox activity and being able to easy interconvert between Fe(II) and Fe(III). In this capacity, Ti(IV) via transmetalation could work to inhibit molecular mechanisms dependent on Fe and its redox activity [202]. A series of Ti(IV) compounds has been made with a family of Fe(III) chelators termed chemical transferrin mimetics (cTfm). These chelators mimic Fe(III) coordination by the blood transport protein serum transferrin, which is responsible for the delivery of Fe(III) into cells. This protein also serves to transport Ti(IV) and binds it in the same binding site as Fe but with some different ligating atoms [203]. The cTfm chelators form very stable Ti(IV) compounds but have a higher affinity for Fe(III) and this preferential binding facilitates transmetalation. Solution studies that approximate Ti(IV) binding conditions in the blood have demonstrated that these compounds remain intact and that only in the intracellular environment are they dissociated by the labile Fe pool [201,202]. The chelator Def (Figure 9) serves as an excellent cTfm representative. Ti(Deferasirox)_2_^2−^ exists at pH 7.4 and it is able to decrease the bioavailability of Fe in Jurkat cells and thereby inhibit the activation of RR [202] similar to findings reported by Tomat et al. and their prochelators [187]. In a recent surprise finding, Ti(Def)_2_^2−^ also transmetalates with Cu(II) possibly leading to the formation of a multinuclear species [Cu(Def)]*_n_^n^*^−^. A metal competition study between labile citrate sources of Fe(III), Cu(II), and Zn(II) with Ti(Def)_2_^2−^ that was monitored by UV-Vis spectroscopy showed that both Cu(II) and Fe(III) underwent transmetalation and Zn(II), which can bind to Def, exhibited no reactivity (Figure 11) [204]. Characteristic ligand to metal charge transfer absorbances were observed for the formation of the [Cu(Def)]*_n_^n^*^−^ and Fe(Def)_2_^3−^ species. Ti(Def)_2_^2−^ has been shown to operate by triggering apoptotic cell death [196]. Correlations with possible Cu and Fe chelation and apoptosis are being further studied. This work elucidates a transmetalation strategy to directly impact both Cu and Fe in cancer cells, which could lead to a very promising drug considering the roles Cu and Fe play in cancer progression.

## 5. Analytical Tools to Quantify and Track Cu and Fe

The study of trace elements and their functions in living organisms and biological systems requires methods to study metals in cells or tissues [205]. This requirement has spawned the field of metallomics [206] and is providing great insight into the biodistribution of metals in the body to understand biomolecular metal regulation, metal accumulation, and abnormalities that could be indicative of a diseased state [201]. The quantification of metals provides information on the biodegradation of biomolecules containing the metals and biodefense patterns due to changes in the levels of the metals [207]. Studies have shown that some patients infected with a bacterial pathogen experience nutritional immunity wherein their blood Felevels purposely decrease to deprive the bacteria of their Fe supply [208]. For purposes of understanding the overall impact of applying Cu and Fe chelators for clinical use, the quantification of Cu and Fe in samples, either in vitro, ex vivo (removing tissue from organisms), or in vivo, is quite useful to determine whether the chelators decrease the levels of these metals in the areas of interest and to what extent can the levels be affected in healthy cells, tissue, blood, etc. Given that not all chelators will necessarily work by directly depleting the metal levels, methods have been devised to try to examine tissue localization and biomolecular binding. This information is often lost during metal quantification due to destructive sample preparation and metal extraction steps. This section will briefly discuss tools that are available for quantifying Cu and Fe and for assessing the Cu and Fe metallome in vitro and in the body.

### 5.1. Techniques to Quantify Cu and Fe Levels

The most common techniques used today for the quantification of Cu and Fe from biological samples in vitro and ex vivo include inductive coupling plasma mass spectrometry (ICP-MS) [209-212], atomic absorption spectrometry (AAS) [212,213], total reflection X-ray fluorescence (TXRF) [214,215], and colorimetric assays by UV-Vis measurements [216,217]. ICP-MS is a highly sensitive tool that allows the precise quantification of metal content down to sub parts per billion (ppb) levels using internal standards [218,219]. These detection limits are possible because highly efficient mass filters and octopolar cells are employed to single out Cu and Fe from possible interferences [220]. The technique is time-consuming, costly, and requires considerable preparation involving sample acid digestion [221]. AAS requires similar sample preparation as ICP-MS but its sensitivity is considerably lower thus requiring higher amounts of samples. In the TXRF technique atoms of the sample (liquids or solids) are excited by a primary X-ray beam and consequently emit low-energy photons that are characteristic of each element [222]. The intensity of each fluorescence radiation is directly proportional to the total amount of the element in the sample after normalization with internal standards [223]. Data collection and processing is fast with this technology and prior sample preparation may not be necessary [224]. The technique is highly sensitive, capable of measuring picograms of Fe [225].

Among the various analytical techniques colorimetry is a simple, reliable, general purpose and cost-effective alternative for routine analysis. While colorimetric assays are easy to perform, sample digestion is often required to release biomolecular bound metal in addition to a specific pH working range. In these assays an absorption spectrum is produced either by a chemical reaction with the metal of interest or from the formation of a colored complex due to ligand binding of the metal. As an example of the latter, the ferrozine-based assay works well to quantify both Fe and Cu [216,217]. Ferrozine (3-(2-pyridyl)-5,6-bis(4-phenylsulfonic acid)-1,2,4-triazine) is an efficient chelator of Fe(II) and Cu(I). To maintain these metals in these oxidations states, reducing agents like hydroxylamine or ascorbic acid are included in the sample preparation [216]. Ferrozine binds to Fe(II) and produces a complex that absorbs strongly at 550 nm [216] whereas the Cu(I) complex absorbs at 470 nm [217]. The ferrozine assay can detect the metals to low ppm values, which is much lower sensitivity compared to ICP-MS (and related techniques), AAS, and TXRF.

### 5.2. Techniques to Track Cu and Fe in Vitro/ex Vivo and in Vivo

There are several techniques available to help localize metals in living cells and tissue in vitro and ex vivo. X-ray absorption spectroscopy (XAS) approaches such as X-ray absorption near-edge structure (XANES) [226], extended X-ray absorption fine structure (EXAFS) [227], and X-ray fluorescence microscopy [228] are quite useful in determining metal speciation in biological samples. However, these tools can be too specialized for practical purposes of measuring metal level differences. Mass spectrometry techniques are more appropriate for these applications such as secondary ion mass spectrometry (SIMS) and laser ablation inductively coupled plasma mass spectrometry (LA-ICP-MS) [229,230]. Electron spectroscopy imaging (ESI) combined with electron energy loss spectroscopy (EELS) have been very useful in monitoring time dependent changes in metal sub-cellular localization [231-233].

A popular approach for visualizing metals especially at low concentrations is the use of metal binding probes via microscopy [170]. An optical signal can be achieved from several sources including fluorescence, phosphorescence, luminescence, or magnetic resonance imaging (MRI) but fluorescence is the most common one. There are three types of fluorescent probes that differ depending on the effect on the fluorescent property that metal binding induces [170]. Intensiometric sensors are those that alter fluorescence intensity after metal binding either resulting in a “Turn-on” or “Turn-off” probe. Ratiometric sensors are those that result in a change in the wavelength of excitation or emission and allow for quantification based on the ratio of wavelength intensities between the metal bound and unbound signals. The third type of probe is the class that changes the fluorescence lifetime after metal binding. These probes can come in the small molecule or macromolecule variety and can be fine-tuned for specific organelle or subcellular localization [170]. To identify a subcellular localization, specific dyes are used such as Propidium Iodide (PI), which labels the nucleus and the dye MitoTracker Red, which labels the mitochondria [234]. Due to the predominance of Cu(I) in the reducing environment of cells, most fluorescent probes have been designed to detect Cu(I). Some probes have been designed by conjugating common Cu chelators with highly fluorescent molecules like BODIPY [234]. For studying Fe in cells, fluorescent probes have been created for Fe(II) and Fe(III). Calcein is commonly used to track Fe(II) [169,170] whereas siderophore-containing probes have been synthesized to study Fe(III) in cells [235].

The field is still quite primitive in tracking metals in the body outside of standard blood testing. MRI has been configured to quantify Fe levels in organs [236]. Chang et al. are leading the field in the development of fluorescent and bioluminescent Cu and Fe probes to study these metals in vivo [237-239]. The clinical application of these techniques is pending.

## 6. Conclusions

Many strategies are being developed to combat the complex set of diseases that constitute cancer. The use of Cu and Fe chelators, while likely not sufficient to combat cancer alone given the status of these metals as essential to the body, holds tremendous promise to be combined with other anticancer approaches. This review has highlighted the numerous ways that Cu and Fe participate in the molecular mechanisms for the onset, proliferation, angiogenesis, and metastasis of cancer. Several Cu and Fe chelators were surveyed to identify important properties that can be exploited in future drug development in addition to structural fine-tuning efforts and analytical tools to improve cellular uptake, cancer cell specificity, and efficacy of the chelators. We anticipate that with the judicial optimization of Cu and Fe chelators and appropriate combination with other drugs that operate in distinct ways, a powerful new treatment will emerge.
